# Synaptotagmin-7 Enhances Facilitation of Ca_v_2.1 Calcium Channels

**DOI:** 10.1523/ENEURO.0081-22.2022

**Published:** 2022-05-12

**Authors:** Alaeddine Djillani, Jeremy Bazinet, William A. Catterall

**Affiliations:** Department of Pharmacology, University of Washington, Seattle, WA 98195-7280

**Keywords:** calcium channels, P/Q-type calcium current, protein interactions, synaptic facilitation, synaptotgmin-7, synprint site

## Abstract

Voltage-gated calcium channel Ca_v_2.1 undergoes Ca^2+^-dependent facilitation and inactivation, which are important in short-term synaptic plasticity. In presynaptic terminals, Ca_v_2.1 forms large protein complexes that include synaptotagmins. Synaptotagmin-7 (Syt-7) is essential to mediate short-term synaptic plasticity in many synapses. Here, based on evidence that Ca_v_2.1 and Syt-7 are both required for short-term synaptic facilitation, we investigated the direct interaction of Syt-7 with Ca_v_2.1 and probed its regulation of Ca_v_2.1 function. We found that Syt-7 binds specifically to the α_1A_ subunit of Ca_v_2.1 through interaction with the synaptic-protein interaction (synprint) site. Surprisingly, this interaction enhances facilitation in paired-pulse protocols and accelerates the onset of facilitation. Syt-7α induces a depolarizing shift in the voltage dependence of activation of Ca_v_2.1 and slows Ca^2+^-dependent inactivation, whereas Syt-7β and Syt-7γ have smaller effects. Our results identify an unexpected, isoform-specific interaction between Ca_v_2.1 and Syt-7 through the synprint site, which enhances Ca_v_2.1 facilitation and modulates its inactivation.

## Visual Abstract

**Figure:**
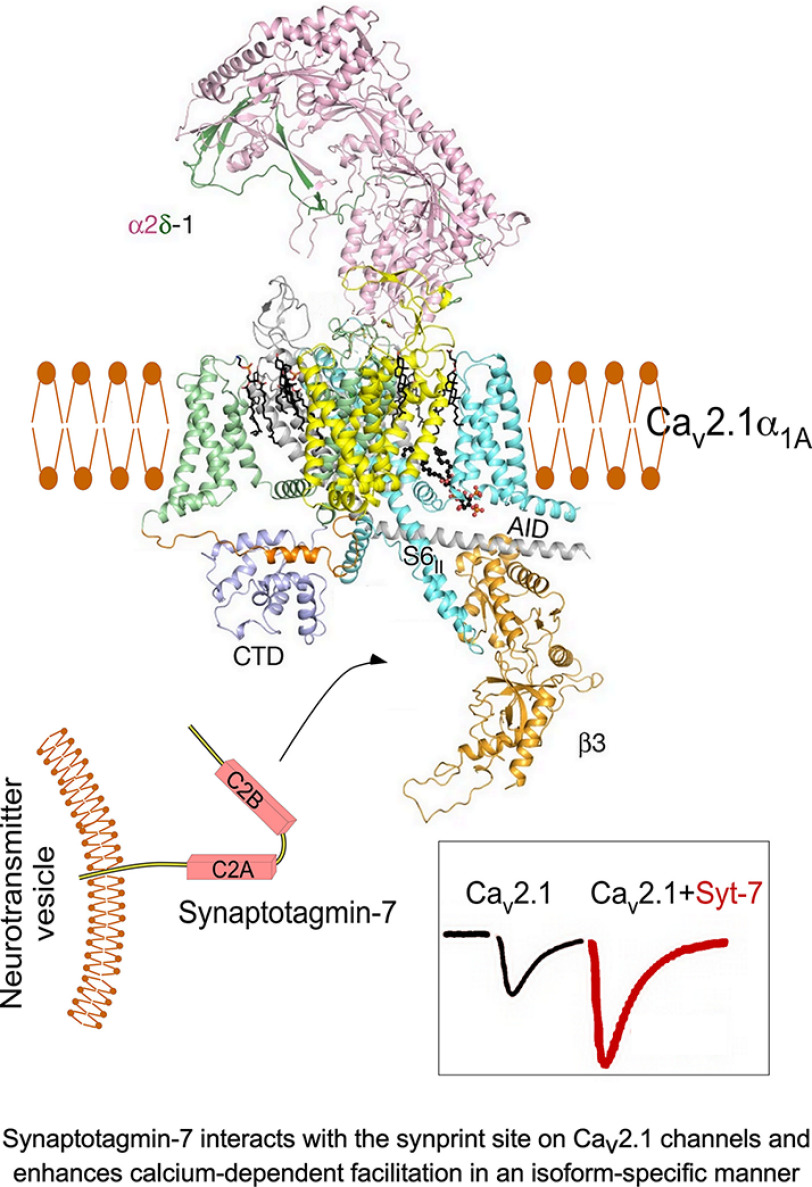

## Significance Statement

Short-term synaptic plasticity mediated by regulation of Ca_v_2.1 channels plays a crucial role in information processing, learning, and memory. Our results reveal a novel mode of regulation of Ca_v_2.1 channels by the high-affinity Ca^2+^ sensor synaptotagmin-7 (Syt-7) through direct interaction with the synprint site. Ca_v_2.1/Syt-7 interaction enhances short-term facilitation of the P/Q-type Ca^2+^ current that triggers neurotransmitter release. This unexpected intersection of Syt-7 and Ca_v_2.1 may regulate short-term, Ca^2+^-dependent synaptic plasticity, along with SNARE proteins and other calcium-binding proteins in presynaptic terminals. Understanding the mechanism by which Syt-7 enhances facilitation of Ca_v_2.1 channels is an important step toward deciphering the molecular mechanisms of short-term synaptic plasticity in the brain.

## Introduction

Inward Ca^2+^ currents conducted by voltage-gated Ca^2+^ (Ca_v_) channels couple action potentials and other depolarizing stimuli to many Ca^2+^-dependent intracellular processes, including neurotransmission, hormone secretion, and muscle contraction (Zamponi et al., 2015). In presynaptic nerve terminals, Ca_v_2.1, Ca_v_2.2, and Ca_v_2.3 channels conduct P/Q-type, N-type, and R-type Ca^2+^ currents that trigger rapid neurotransmission (for review, see Olivera et al., 1994; Zamponi et al., 2015; Nanou and Catterall, 2018). However, only P/Q-type Ca^2+^ currents conducted by Ca_v_2.1 channels can mediate short-term synaptic facilitation at the calyx of Held in mice (Inchauspe et al., 2004), pointing to a unique role of these Ca^2+^ channels in short-term synaptic plasticity.

In transfected nonneuronal cells, Ca^2+^ entry mediated by Ca_v_2.1 channels causes calcium-dependent facilitation (CDF) and inactivation (CDI) during single depolarizations and in trains of repetitive depolarizing pulses (Lee et al., 1999, 2000; DeMaria et al., 2001; Catterall and Few, 2008; Christel and Lee, 2012; Ben-Johny and Yue, 2014). Both CDF and CDI of Ca_v_2.1 channels are dependent on calmodulin (CaM; Lee et al., 1999, 2000; DeMaria et al., 2001). CaM preassociates with the C-terminal domain of the pore-forming α1 subunit of Ca_v_2.1 channels (Erickson et al., 2001). Following Ca^2+^ binding, CaM initially interacts with the nearby IQ-like motif (IM) and causes CDF, whereas further binding of Ca^2+^/CaM to the more distal CaM-binding domain (CBD) induces CDI of Ca_v_2.1 channels (DeMaria et al., 2001; Lee et al., 2003). Introducing the IM-AA mutation into the IQ-like motif of Ca_v_2.1 impairs CDF and CDI, providing a tool to assess the significance of these processes in synaptic transmission and short-term synaptic plasticity (Zühlke et al., 1999; DeMaria et al., 2001; Lee et al., 2003).

CDF of Ca_v_2.1 channels contributes significantly to short-term synaptic facilitation. Expression of Ca_v_2.1 in cultured superior cervical ganglion neurons, whose endogenous Ca_v_2.2 channels were specifically blocked by ω-conotoxin GVIA, was sufficient to restore synaptic transmission and induce Ca^2+^-dependent synaptic facilitation, which was impaired by introducing the IM-AA mutation in Ca_v_2.1 channels (Mochida et al., 2003a, 2008). In mice in which Ca_v_2.1 channels contained the IM-AA mutation, synaptic facilitation was substantially decreased at the neuromuscular junction as well as in hippocampal CA3-to-CA1 synapses and CA3-to-parvalbumin-expressing basket cell synapses (Nanou et al., 2016a,b, 2018). These results support an important role for facilitation of Ca_v_2.1 channels in short-term synaptic facilitation.

In addition to Ca_v_2.1 channels, the high-sensitivity Ca^2+^ sensor synaptotagmin-7 (Syt-7) has been proposed to support short-term synaptic facilitation by binding residual Ca^2+^ in the nerve terminal following the action potential, thereby increasing interaction with the SNARE complex and enhancing Ca^2+^-dependent synaptic vesicle exocytosis (Jackman et al., 2016). Previous studies using Syt-7 KO mice have shown that Syt-7 is required for short-term plasticity in several types of synapses in the hippocampus, cerebral cortex, and cerebellum (Jackman et al., 2016; Turecek and Regehr, 2018). Because Ca_v_2.1 channels and Syt-7 are located near each other in the active zones of nerve terminals (Müller et al., 2010), and Ca_v_2.1 and Syt-7 are both implicated in synaptic facilitation, we have tested the hypothesis that these two proteins interact directly with each other and regulate Ca^2+^ entry through Ca_v_2.1 channels. Our results reveal direct interactions of Syt-7 with Ca_v_2.1 that enhance facilitation of the Ca_v_2.1 Ca^2+^ current. These data suggest that interaction of Syt-7 with Ca_v_2.1 channels may contribute to short-term synaptic facilitation.

## Materials and Methods

### Cell lines and transfection

Cells from tsA-201 cell line were maintained in DMEM (Invitrogen by Life Technologies) supplemented with 10% fetal bovine serum (Fisher Scientific), 1% glutamine (Sigma-Aldrich), 1% penicillin and streptomycin (Sigma-Aldrich). The cells were maintained at 37°C under 5% CO_2_. Cells were plated in 35-mm tissue culture dishes to achieve 70% confluency and then transfected using TransIT-LT1 transfection reagent (Mirus) with a total of 5-μg plasmid including: 2, 1.5, 1 μg of α_1A_, β_2A_, and α_2_δ subunits composing the Ca_v_2.1 channel and a ratio 3 μl of transfection reagent to 1 μg of cDNA plasmid. 0.22 μg eGFP was added to the plasmid mix to identify the transfected cells.

### Construction and expression of fusion proteins

Recombinant glutathione S-transferase (GST)-Syt-7α fusion proteins were synthesize from the expression plasmid in the vector pGEX-2T. His-fusion proteins containing the synprint site region from the intracellular loop between domain II and III of the P/Q-type Ca_v_2.1 (synprint 724–981) or the equivalent synprint site from the L-type Ca_v_1.2 (680–800) used as a control, were expressed using the expression plasmid pET-28b. GST and His recombinant proteins were expressed in *Escherichia coli* BL26 cells, a protease-deficient strain (NEB). Fusion proteins were extracted by mild sonication (10 times 10 s with 1-min break) in lysis buffer containing: Tris 50 mm (pH 7.4), NaCl 150 mm, Na-deoxycholate 1%, NaF 10 mm, EDTA 1 mm, Triton X-100 1%, and glycerol 5%, supplemented with protease inhibitors Calpain I, Calpain II, and cOmplete protease inhibitor cocktail (Sigma-Aldrich). GST-Syt-7α, proteins were purified using glutathione Sepharose beads (Millipore Sigma) and eluted with 15 mm reduced glutathione (GSH) in 50 mm Tris (pH). His-synprints from Ca_v_2.1 and Ca_v_1.2 were purified by binding to Ni^2+^-charged HisPur Ni-NTA Resin (ThermoFisher) and eluted with 250 and 500 mm imidazole in PBS. The amount of proteins used was standardized based on Coomassie Blue-stained SDS gels or estimated with a standard curve relating the intensity of the immunoblotting signal to the amount of a standard fusion protein applied.

### Co-immunoprecipitation experiments

Immunoprecipitation experiments were performed using Dynabeads Protein G (Invitrogen) in TBS buffer with a Ca^2+^-buffering system containing 50 mm Tris/HCl, 140 mm NaCl, 50 mm HEPES (pH 7.2), 5 mm N-(2-hydroxyethyl)ethylenediamine-N,N′,N′-triacetic acid (HydroxyEDTA), 0.3% Triton X-100 and different Ca^2+^ concentrations varying from 10 μM to 5 mM. The Ca^2+^-buffering system was used to produce free Ca^2+^ concentrations calculated using MAX CHELATOR software (UC Davis). Dynabeads were incubated with antibodies directed against Ca_v_2.1 channels or Syt-7α for 1 h at 4°C. Then, whole brain lysates or transfected tsA cell lysates were added to the beads and incubated at 4°C under rotation overnight. Nonspecific proteins were washed three times with a washing buffer. Proteins attached to the beads were eluted using an elution buffer. Proteins were blotted with antibodies against Syt-7 (mouse monoclonal antibody N275/14, Product Number MABN665, Millipore Sigma) or Ca_v_2.1 (rabbit polyclonal antibody catalog #ACC-001, Alomone Labs). The antibodies used for immunoblotting were titrated to assure that the concentration used was in the linear response range. The co-immunoprecipitation experiments and western blots have been repeated at least three times showing reproducible results.

### Study of Syt-7 binding to the synprint site by affinity chromatography

GST-Syt-7α proteins were bound to glutathione-Sepharose beads (Millipore Sigma) in TBS-Ca^2+^ buffer incubated at 4°C for 1 h under constant rotation. To remove unbound proteins, the mixture was washed two times with a washing buffer. Glutathione-Sepharose beads coupled with GST-fusion proteins were added to similar amount of purified His-Ca_v_2.1 synprint (724–981) or His-Ca_v_1.2 synprint (680–800). The mixture was incubated under constant rotation for 1 h at 4°C. The binding experiments were conducted in presence of TBS-Ca^2+^ buffering system with 0.1% Triton X-100. The beads were washed three times with washing buffer and bound complexes were eluted with 15 mm of reduced glutathione and 50 mm Tris-HCl (pH 8). Eluates were separated from beads by centrifugation at 10,000 × *g* for 1 min and processed for 10–20% SDS/tricine gradient gel electrophoresis and immunoblotted with anti-His antibody.

### Electrophysiological recording

Calcium current (*I*_Ca_) or Barium current (*I*_Ba_) were recorded at least 48 h after tsA-201 cell transfection using whole-cell configuration of the patch-clamp technique. Data acquisition was conducted using patch-clamp amplifier (HEKA Elektronik GmbH). Voltage-clamp protocols and facilitation protocols were applied, and data were acquired using Pulse (HEKA Elektronik GmbH). Currents were filtered at 5 kHz. Leak and capacitance transient currents were subtracted using a P/4 protocol.

Recording pipettes were pulled from borosilicate glass to achieve initial bath resistances of 1.5–3.0 MΩ and filled with an intrapipette solution containing (in mm): 120 *N*-methyl-D-glucamine (NMDG), 60 HEPES, 1 MgCl_2_, 2 Mg-ATP, and 0.5 EGTA. The extracellular patch-clamp solution contained (in mm): 150 Tris, 1 MgCl_2_, and 10 CaCl_2_ or BaCl_2_ depending on the experimental protocols. The pH of both intrapipette and extracellular solutions was adjusted to 7.3 using methanesulfonic acid. tsA-201 cell membrane capacitance (Cm) varied from 15–25 pF and access resistance (Rs) varied from 8 to 20 MΩ. All averaged data represent the mean ± SEM of at least 10 cells. For peak current measurement using current–voltage (I/V) curves, Ca_v_2.1 P/Q-type current was generated using steps of depolarization from −80 to +60 mV every 10-mV step with a holding potential at −80 mV. A total of 10 mm CaCl_2_ or BaCl_2_ was used in the external patch-clamp solution. For facilitation protocol experiments, 10 mm of CaCl_2_ were used in the external solution. Several facilitation protocols have been used to study the role of the three isoforms of Syt-7 (Syt-7α, Syt-7β, and Syt-7γ) in Ca^2+^ current facilitation. Paired-pulse facilitation protocols were evoked by applying two 1-s-spaced depolarizing pulses P1 and P2 from −80 to +10 mV. A preconditioning 50-ms depolarizing step from −80 to +10 mV was applied only 5 ms before P2. In order to study the voltage dependence of Ca_v_2.1 channel activation, P1 and P2 were applied using variable voltages from −120 to +40 mV. P2 over P1 ratios were calculated and compared between transfected tsA-201 cells with and without Syt-7α, Syt-7β, or Syt-7γ. The second protocol of paired-pulse facilitation was used to study the effect of changing voltages in the preconditioning pulse on P2. P1 and P2 pulses were maintained from −80 to +10 mV; however, the preconditioning pulse was applied with variable voltages from −120 to +40 mV. Finally, onset of facilitation was studied by increasing preconditioning pulse duration to 10 ms and measuring the ratios of P2 over P1.

### Statistical analysis

Statistical analyses were performed using GraphPad Prism 5 (GraphPad Software) and Origin Pro (OriginLab Inc.). All data are shown as the mean ± SEM. The statistical details of the experiments can be found the results section and figure legends. A Student’s *t* test was used to compare two sets of data. The significance was defined using a threshold of *p* = 0.05 throughout the study. Error bars indicate SEM. Sample sizes are described in the figure legends.

## Results

### Syt-7 binds to the Ca_v_2.1 channel in mouse brain

**Figure 1.**
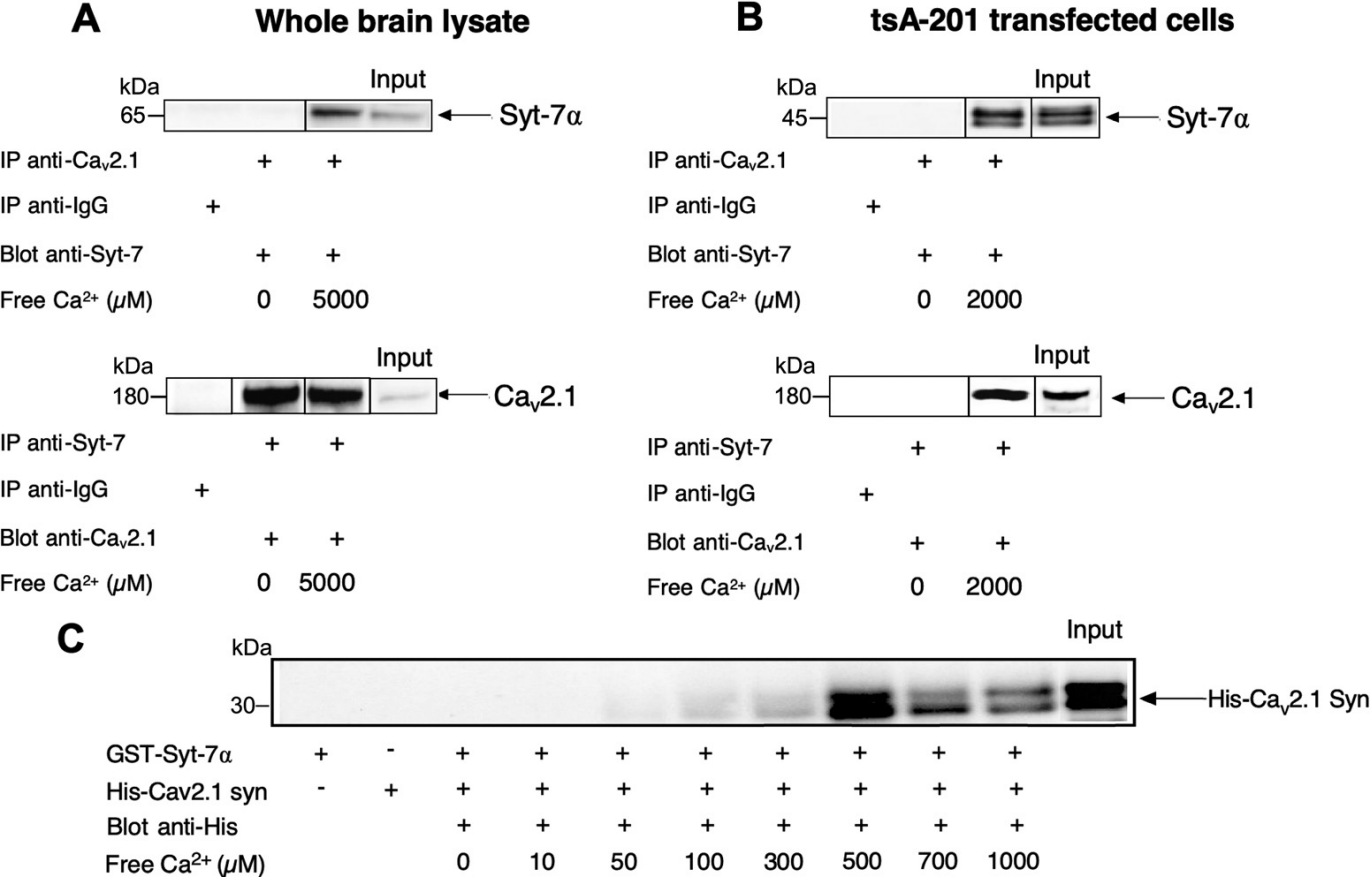
Direct binding of Syt-7α to the synprint site of P/Q-type Ca_v_2.1 channels. ***A***, ***B***, In brain lysates (***A***) and transfected tsA-201 cells (***B***), co-immunoprecipitation experiments show the binding of Syt-7α and α_1A_ subunit of Ca_v_2.1 channels**. *C***, Binding of Syt-7α (GST-Syt-7α) to the His-tagged synprint site (724–981) of α_1A_ subunit of P/Q-type Ca_v_2.1 channel. GST-Syt-7α were immobilized on glutathione-Sepharose beads and incubated with His-Ca_v_2.1 or His-Ca_v_1.2 using different Ca^2+^ concentrations (10 μm to 1 mm). The binding experiments were performed in a Ca^2+^ buffering system containing: 5 mm N-hydroxyethyl ethylenediaminetriacetic, 50 mm HEPES (pH 7.2), and 150 mm NaCl. The free Ca^2+^ concentrations were estimated using the MAX CHELATOR software. The Ca^2+^ concentrations used were 10 μm, 50 μm, 100 μm, 300 μm, 500 μm, 700 μm, and 1 mm. After extensive washing, GST-Syt-7α bound to the beads were eluted with 15 mm reduced glutathione (GSH) in 50 mm Tris-HCl (pH 8) and proceed to electrophoresis and immunoblotting. The bound His-Ca_v_2.1 synprint (724–981) proteins were detected with anti-His antibody. Because different Ca^2+^ concentrations were used in co-immunoprecipitation experiments, segments from different immunoblots were spliced together to show comparisons clearly. Those protein bands are delineated for clarification. The immunoblots presented here are representative of at least three experiments for each co-immunoprecipitation or immunoblot. A related co-immunoprecipitation experiment conducted with different experimental conditions is presented in Extended Data Figure 1-1.

To determine whether the slow, high-affinity Ca^2+^ sensor Syt-7 binds to the presynaptic Ca_v_2.1 channels *in vivo*, co-immunoprecipitation studies were performed on membrane preparations from mouse brain lysates. Ca_v_2.1 channels extracted from these neuronal membranes were immunoprecipitated with specific anti-Ca_v_2.1 antibodies, and the resulting complexes were probed with anti-Syt-7 antibody using the Dynabeads/Protein G co-immunoprecipitation protocol. The resulting immunoblots revealed Ca_v_2.1/Syt-7α interaction with anti-Syt-7 when anti-Ca_v_2.1 was used as the precipitating antibody (Fig. 1*A*, top). In a complementary experiment, Ca_v_2.1 channels were immunoprecipitated with anti-Syt-7 antibodies and detected in immunoblots with anti-Ca_v_2.1 antibodies (Fig. 1*A*, bottom). Co-immunoprecipitation experiments using a modified experimental protocol yielded comparable results (Extended Data Fig. 1-1). Together, these results show that Ca_v_2.1 channels and Syt-7 are associated with each other in mouse brain membranes.

10.1523/ENEURO.0081-22.2022.f1-1 Extended Data Figure 1-1 Co-immunoprecipitation of Syt-7 and Syt-1 with Cav2.1 channels. A, In mouse brain lysate, Syt-7 proteins co-immunoprecipitated with Cav2.1 channels in three different buffered concentrations of free Ca2+. B, Co-immunoprecipitation of Syt-7 and Syt-1 with Cav2.1 from co-transfected tsA-201 cells. Syt-7 is pulled down by Cav2.1-specific antibodies in Cav2.1 and Syt-7 co-transfected tsA-201 cell lysates. Download Figure 1-1, TIF file.

### Syt-7 binds to the synprint site of the Ca_v_2.1 channel

Human embryonic kidney tsA-201 cells were transfected with the α_1A_, β_2A_, and α_2_δ_1_ subunits of Ca_v_2.1 channels together with Syt-7α, the most abundant isoform of Syt-7 (Fukuda et al., 2002). A specific complex of Ca_v_2.1 and Syt-7 was co-immunoprecipitated from lysates of tsA-201 cells transfected with Ca_v_2.1α_1A_ subunit and Syt-7α, using either anti-Syt-7 or anti-Ca_v_2.1 as the precipitating antibody (Fig. 1*B*). These results demonstrate a physical interaction between Syt-7α and Ca_v_2.1 in intact cells expressing these proteins *in vitro*, and suggest that other neuron-specific proteins are not required for this protein-protein interaction.

To investigate which domain of the pore-forming α_1_ subunit of Ca_v_2.1 channels binds Syt-7α, *in vitro* binding experiments were performed using recombinant fusion proteins (Materials and Methods). Full-length Syt-7α protein was expressed as a GST-fusion protein, and the synprint site in Ca_v_2.1 (724–981) was expressed as a His-fusion protein. As a control, the equivalent synprint site from the cardiac Ca^2+^ channel, Ca_v_1.2 (680–800), was expressed as a His-fusion protein. GST-Syt-7α proteins were immobilized by binding to glutathione-Sepharose beads and incubated with a constant concentration of His-Ca_v_2.1 synprint peptide (724–981) or His-Ca_v_1.2 synprint peptide (680–800) using different free Ca^2+^ concentrations varying from 10 μm to 1 mm. After extensive washing, binding of His-Ca_v_2.1 synprint (724–981) to GST-Syt-7α was revealed by immunoblot analysis using an anti-His antibody. As shown in Figure 1*C*, GST-Syt-7α bound to His-Ca_v_2.1 (724–981) synprint in a Ca^2+^-dependent manner *in vitro*, with binding first detected at 50 μm free Ca^2+^ concentration and increasing to a maximum at 500 μm Ca^2+^. In contrast, the negative control peptide His-Ca_v_1.2 (680–800) from the corresponding segment of cardiac Ca_V_1.2 channels did not bind to GST-Syt-7α. These results demonstrate specific binding of Syt-7 to the synprint site from Ca_v_2.1 channels in preference to the corresponding segment of the cardiac Ca_v_1.2 channel.

### Syt-7α increases the rate and extent of Ca^2+^-dependent facilitation

**Figure 2.**
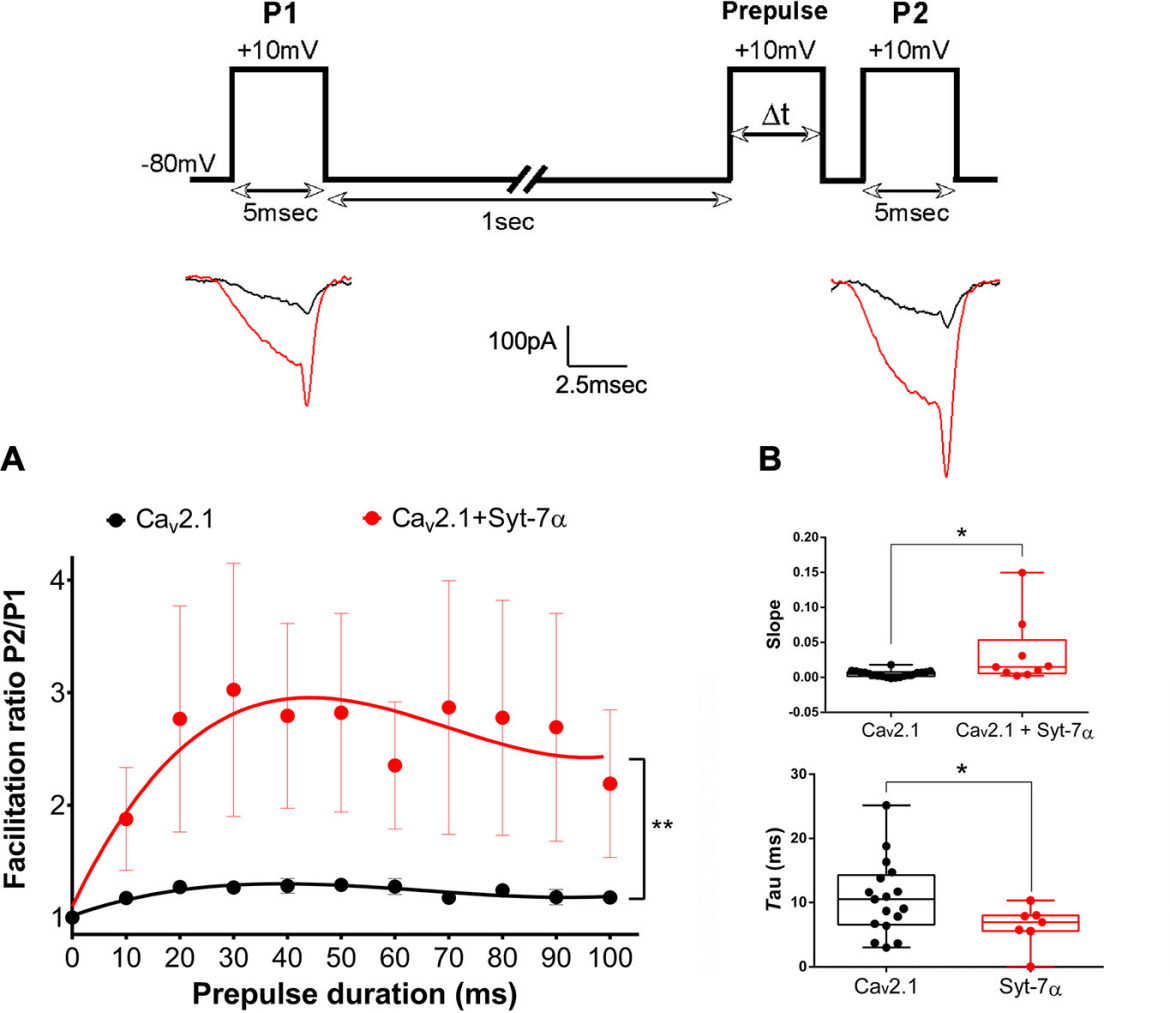
Syt-7α accelerates the onset of facilitation of Ca_v_2.1 channels. Inset top, Pulse protocol. Currents recorded with 10 mm extracellular Ca^2+^ and 0.5 mm EGTA in the intracellular recording solution were elicited by test pulses to +10 mV before (P1) and 5 ms after (P2) 10-mV preconditioning prepulses of the indicated durations. Inset, Example traces from control and Syt-7α transfected tsA cells following P1 and P2 pulses. ***A***, Effect of Syt-7α on facilitation as a function of prepulse duration. Facilitation was obtained by normalizing the peak current from P2 to that from P1. Single-exponential fits of the data are shown. ***B***, in tsA-201 cells co-expressing Ca_v_2.1 channel with Syt-7α, the slope is significantly increased compared with control cells. Data are represented as mean ± SEM.

Previous studies have shown the key role of Ca_v_2.1 channels (Lee et al., 2000; Mochida et al., 2003a, 2008; Inchauspe et al., 2004) and Syt-7 (Jackman et al., 2016; Turecek and Regehr, 2018) in synaptic facilitation, but it is not known whether functional interactions between these two proteins modulate paired-pulse facilitation of P/Q-type Ca^2+^ currents using pulse protocols similar to those in studies of short-term synaptic facilitation. In order to characterize the mechanism by which Syt-7α increases Ca^2+^-dependent facilitation, the effects of Syt-7α on the onset and decay of facilitation were measured with 10 mm Ca^2+^ in the external solution to mimic the high local Ca^2+^ concentration near the intracellular mouth of Ca_v_2.1 channels in nerve terminals during synaptic transmission. In a paired-pulse protocol, the rate of onset of facilitation was determined by plotting facilitation of *I*_Ca_ as a function of prepulse duration (Δt; Fig. 2, inset). In cells expressing only Ca_v_2.1 channels, the facilitation ratio increased with prepulse duration according to a single-exponential time course (Fig. 2*A*, black). Facilitation ratio reached a plateau at a prepulse duration of 20 ms and declined during prepulses of 50 ms or longer (P2/P1 ratio = 1.29 ± 0.06, *n* = 20). In tsA-201 cells co-expressing Ca_v_2.1 channels with Syt-7α, facilitation increased more rapidly, reached a higher plateau at prepulse durations of 20–30 ms, and declined slowly during prepulses with Δt > 30 ms (Fig. 2*A*,*B*, red; P2/P1 ratio = 3.03 ± 1.13, *n* = 9). The increase in facilitation ratio was significant at all prepulse durations compared with control cells (*p* < 0.0001; Fig. 2*A*,*B*). The rate of increase in facilitation was significantly steeper in cells co-expressing Ca_v_2.1 with Syt-7α (slope = 0.03 ± 0.02 ms^−1^; *n* = 9, *p* = 0.01) compared with control cells (slope = 0.005 ± 0.001 ms^−1^; *n* = 20; Fig. 2*A*,*B*, red), and the half-time was shorter (τ = 6.37 ± 1.22 ms, *n* = 7, *p* = 0.03) compared with control cells (τ = 10.75 ± 1.41 ms, *n* = 17; Fig. 2*A*,*B*, red). Taken together, these data show that Syt-7α increases the rate of the onset of facilitation of Ca_v_2.1 channels and increases the facilitation ratio at all prepulse durations tested.

**Figure 3.**
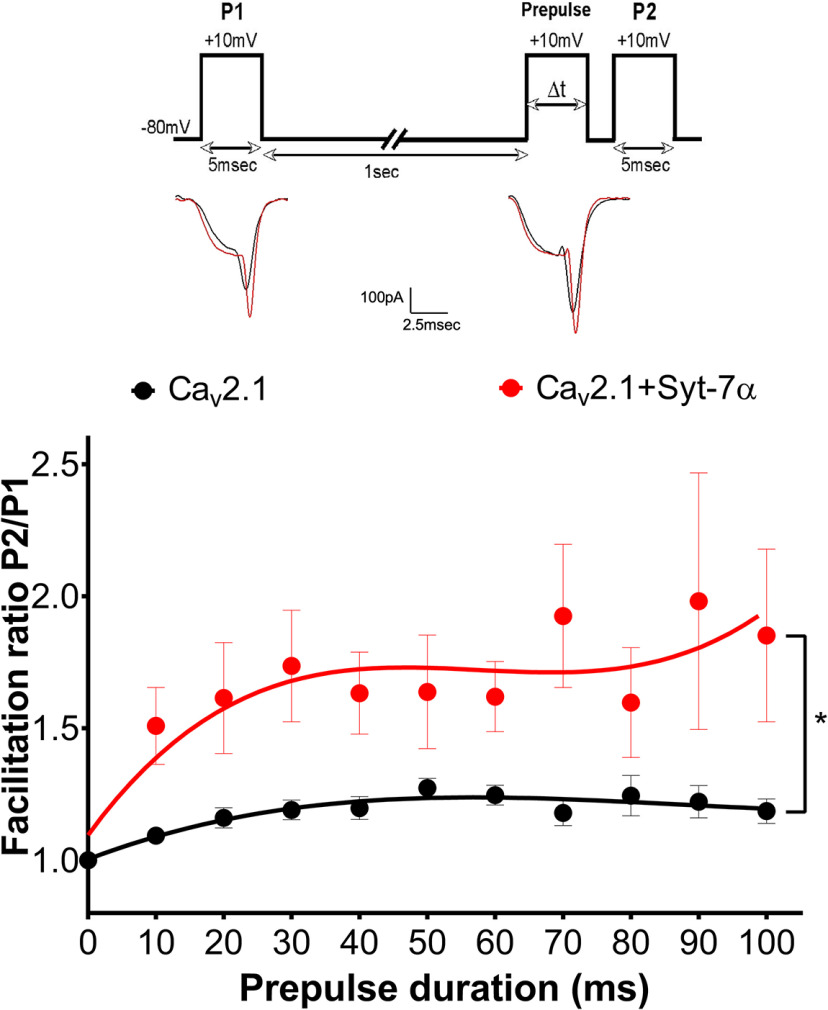
Effect of Syt-7α on prepulse facilitation of Ca_v_2.1 at physiological Ca^2+^ levels. Inset top, Pulse protocol. Currents recorded with 2 mm extracellular Ca^2+^ and 0.5 mm EGTA in the intracellular recording solution were elicited by test pulses to +10 mV before (P1) and 5 ms after (P2) 10-mV conditioning prepulses of the indicated durations. Inset, Example traces from control and Syt-7α transfected tsA cells following P1 and P2 pulses. Main panel, Graph shows the effect of Syt-7α on facilitation as a function of prepulse duration. Facilitation was obtained by normalizing the peak current from P2 to that from P1. Single-exponential fits of the data are shown. Data are represented as mean ± SEM.

Intracellular Ca^2+^ concentrations near presynaptic Ca^2+^ channels rise to nearly 100 μM during rapid stimulation (Berridge et al., 2000). To mimic that condition, we have used 10 mm extracellular Ca^2+^ in our standard experimental protocol to generate high Ca^2+^ influx. However, at the physiological level of extracellular Ca^2+^, with 2 mm CaCl_2_ the external recording solution, applying paired-pulse protocols revealed a significant acceleration of the onset and increase of the extent of Ca^2+^-dependent facilitation in cells co-expressing Ca_v_2.1 combined with Syt-7α compared with control (Fig. 3), as we observed with 10 mm external Ca^2+^ concentration.

### Syt-7α induces a rapidly decaying form of Ca^2+^-dependent facilitation

**Figure 4.**
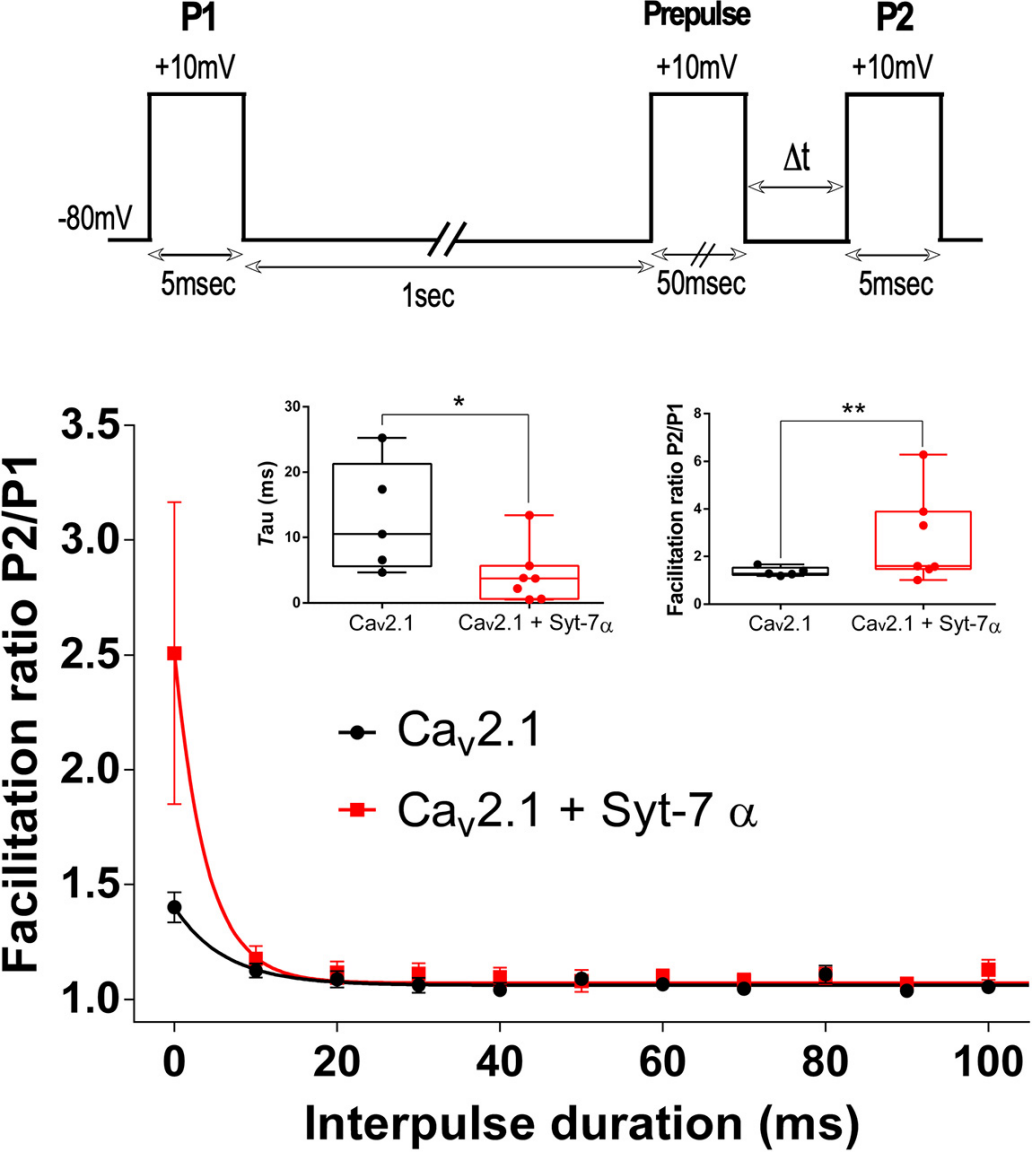
Effect of Syt-7α on the decay from facilitation of Ca_v_2.1 channels. Inset top, Pulse protocol for measuring decay of facilitation. Ca^2+^ currents are elicited by test pulses to +10 mV before (P1) and after (P2) a conditioning prepulse to +10 mV for 5 ms. Inset bottom left, Decay from facilitation measured by comparing τ between control and Syt-7α transfected cells. Inset bottom right, Comparison of P2/P1 facilitation ratio at Δ*t* = 0 between control and Syt-7α-expressing cells. Main panel. Effect of Syt-7α on the decay from facilitation. The facilitation ratio was obtained by normalizing the peak current from P2 to that from P1 and was plotted against the interval between the conditioning prepulse and P2. Shown are results obtained with 50-ms conditioning prepulse. Graph shows the effect of Syt-7α on decay of facilitation as a function of interpulse duration. Data are represented as mean ± SEM.

Syt-7α accelerates the onset of *I*_Ca_ facilitation and increases facilitation amplitude at all potentials. However, the increased facilitation caused by Syt-7α decayed rapidly (τ = 4.29 ± 1.68 ms, *p* = 0.02, *n* = 7), compared with facilitation of Ca_v_2.1 observed for tsA-201 cells in the absence of Syt-7α (τ = 12.87 ± 3.77 ms, *n* = 5; Fig. 4, inset, left). The facilitation ratio P2/P1 at the first interpulse duration point was significantly greater for Ca_v_2.1-Syt-7α cells (facilitation ratio = 2.73 ± 0.71, *p* = 0.004, *n* = 7) than for control cells (facilitation ratio = 1.36 ± 0.08, *n* = 5; Fig. 4, inset, right). Although the facilitation ratio in the presence of Syt-7α decays rapidly, the integral of calcium current during the first 10 ms following stimulation is substantially increased (Fig. 4), illustrating the potential physiological significance of this increase in Ca_v_2.1 channel activity.

### Syt-7α increases voltage-dependent facilitation in paired-pulse protocols

**Figure 5.**
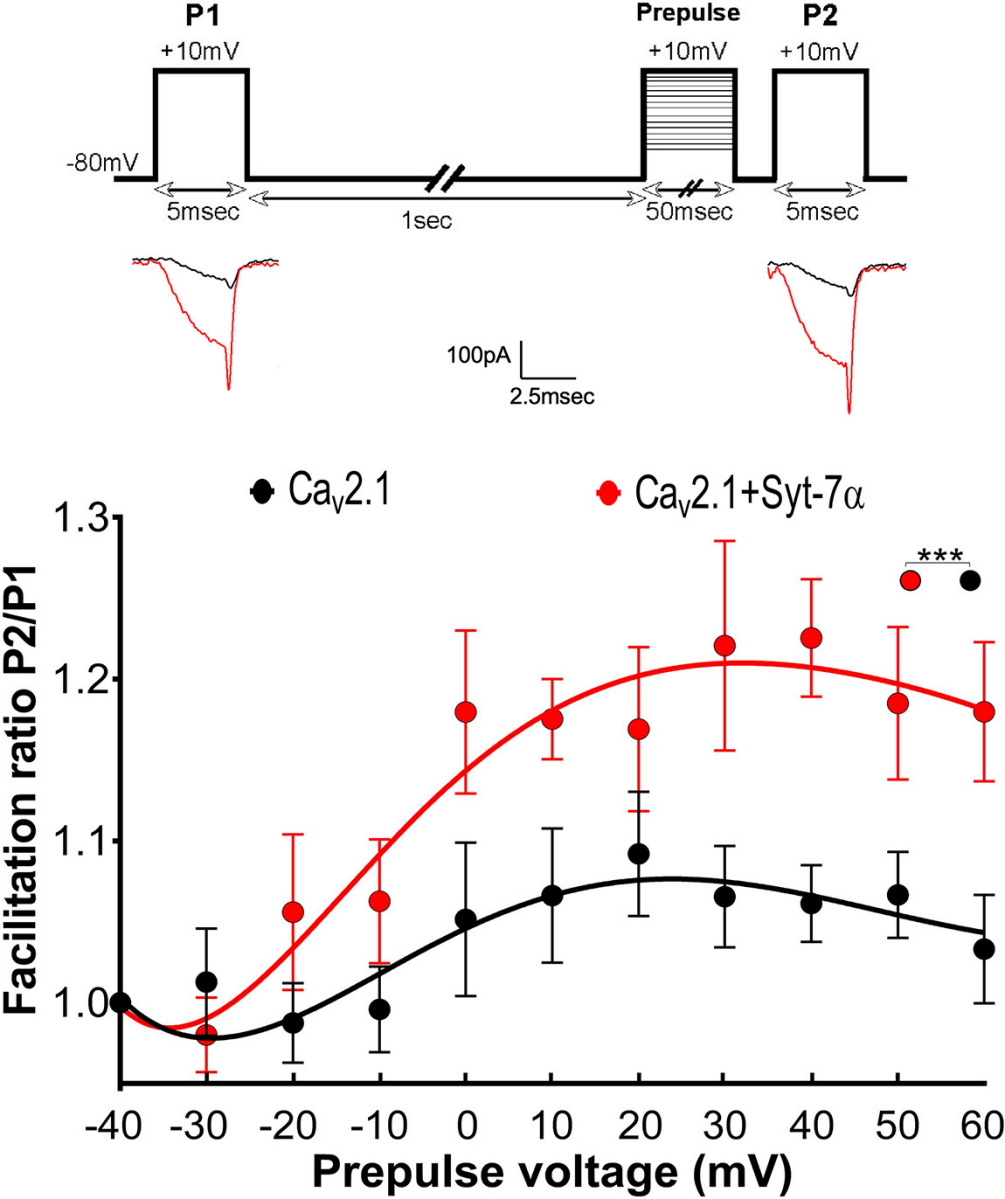
Syt-7α potentiates Ca_v_2.1 facilitation in a paired-pulse protocol following change in prepulse voltage. Inset top, Pulse protocol shown represents paired pulse protocol. Ca^2+^ current was recorded using 10 mm Ca^2+^ and 0.5 mm EGTA in the external and internal solutions, respectively. Pulse 1 (P1; depolarization from −80 to +10 mV) elicits the first Ca^2+^ current. A second 5-ms pulse (P2) generating a second I_Ca_ is applied 2 ms after a 50-ms conditioning prepulse with variable voltages (−40 to 60 mV). Inset bottom, Example traces from control and Syt-7α transfected tsA cells following P1 and P2 pulses. Main panel, Graph shows the effects of Syt-7α isoform on facilitation as a function of prepulse voltage. The facilitation ratio was obtained by normalizing the peak current from P2 to that from P1. Data are represented as mean ± SEM.

In order to study the effect of Syt-7 on the voltage dependence of Ca_v_2.1 activation and its consequences on facilitation, we measured facilitation using a paired-pulse protocol with variable stimulus potentials. In this protocol, facilitation induced by a 50-ms-long prepulse to a variable voltage (−40 to +60 mV) was measured by comparing *I*_Ca_ elicited by a test pulse before (P1) and after (P2) the conditioning prepulse (Fig. 5, inset). In cells expressing Ca_v_2.1 alone, paired-pulse facilitation increased to a maximum at a prepulse voltage of +20 mV and remained at a plateau until +60 mV (facilitation ratio at 20 mV =1.09 ± 0.04, *n* = 8; Fig. 5). Co-expression of Ca_v_2.1 with Syt-7α increased the maximum paired-pulse ratio to 1.2 ± 0.05 (*n* = 8 at +40 mV, *p* < 0.01), approximately doubling the increase in Ca^2+^ current induced by paired-pulse facilitation in the absence of Syt-7.

**Figure 6.**
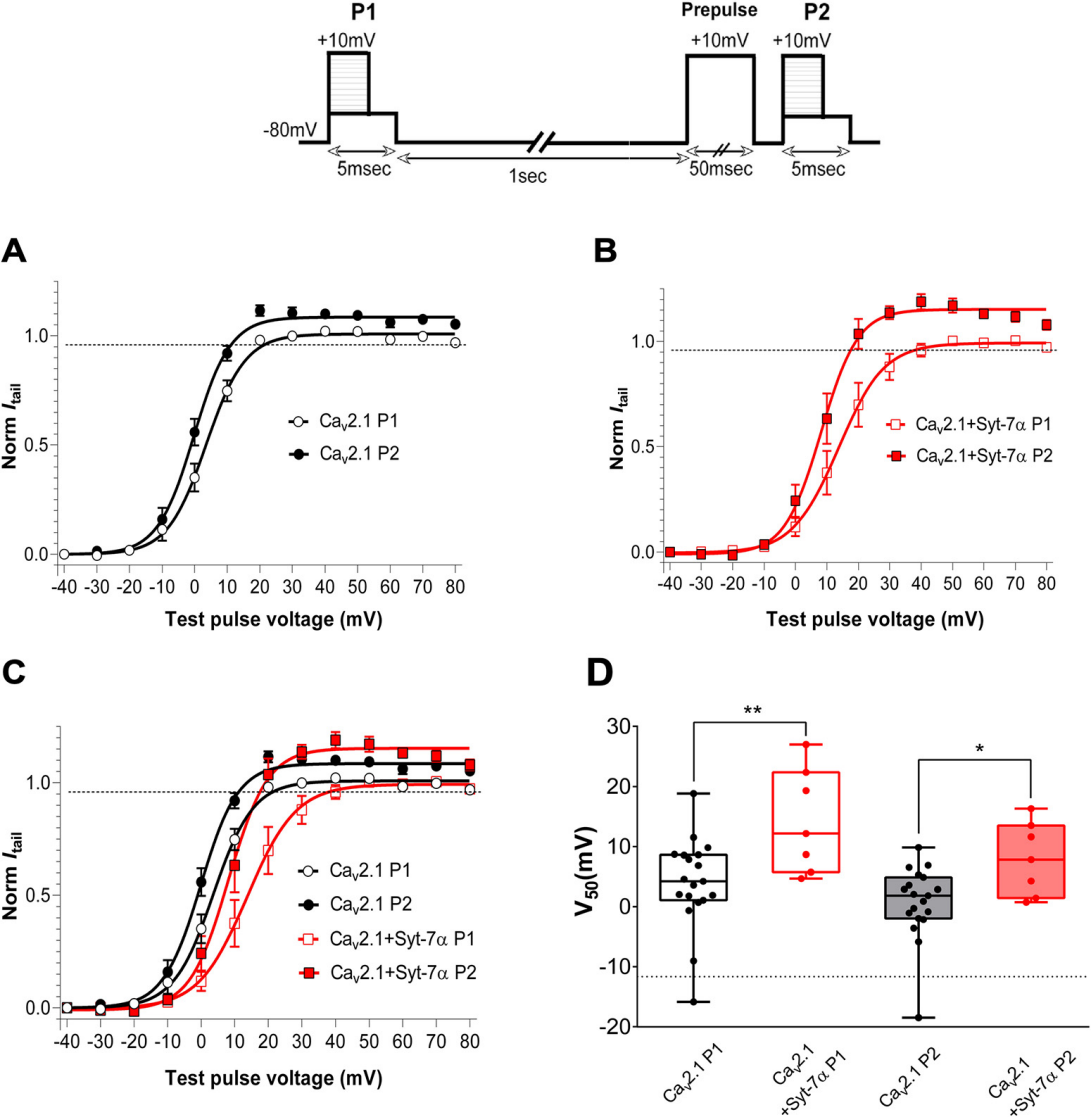
Effect of Syt-7α on prepulse facilitation of Ca_v_2.1 channel. Facilitation of voltage-dependent activation of Ca^2+^ currents. Inset, Pulse protocol to study the voltage dependence of activation before (open circle or squares; P1) and after (closed circle or squares; P2) a depolarizing prepulse from −80 to +10 mV. Tail currents were measured by holding potential at −40 mV for 5 ms after test pulses (P1, P2) to variable voltages (−40 to +80 mV). Peak tail currents were normalized to the largest tail current measured during the nonfacilitated prepulses (P1) and plotted against the test pulse voltage. ***A***, In control tsA cells, the protocol shows an increase in facilitation P2 normalized to P1. ***B***, Syt-7α potentiated facilitation amplitude of Ca_v_2.1 and induced a right shift in prepulse facilitation curve. ***C***, Overlaying the two graphs in ***A***, ***B*** shows the increase in amplitude of facilitation and the right shift in voltage dependency of activation. ***D***, Difference in voltage shift in P1 and P2 between cells co-expressing Ca_v_2.1 and Syt-7α and control cells. Data are represented as mean ± SEM.

**Figure 7.**
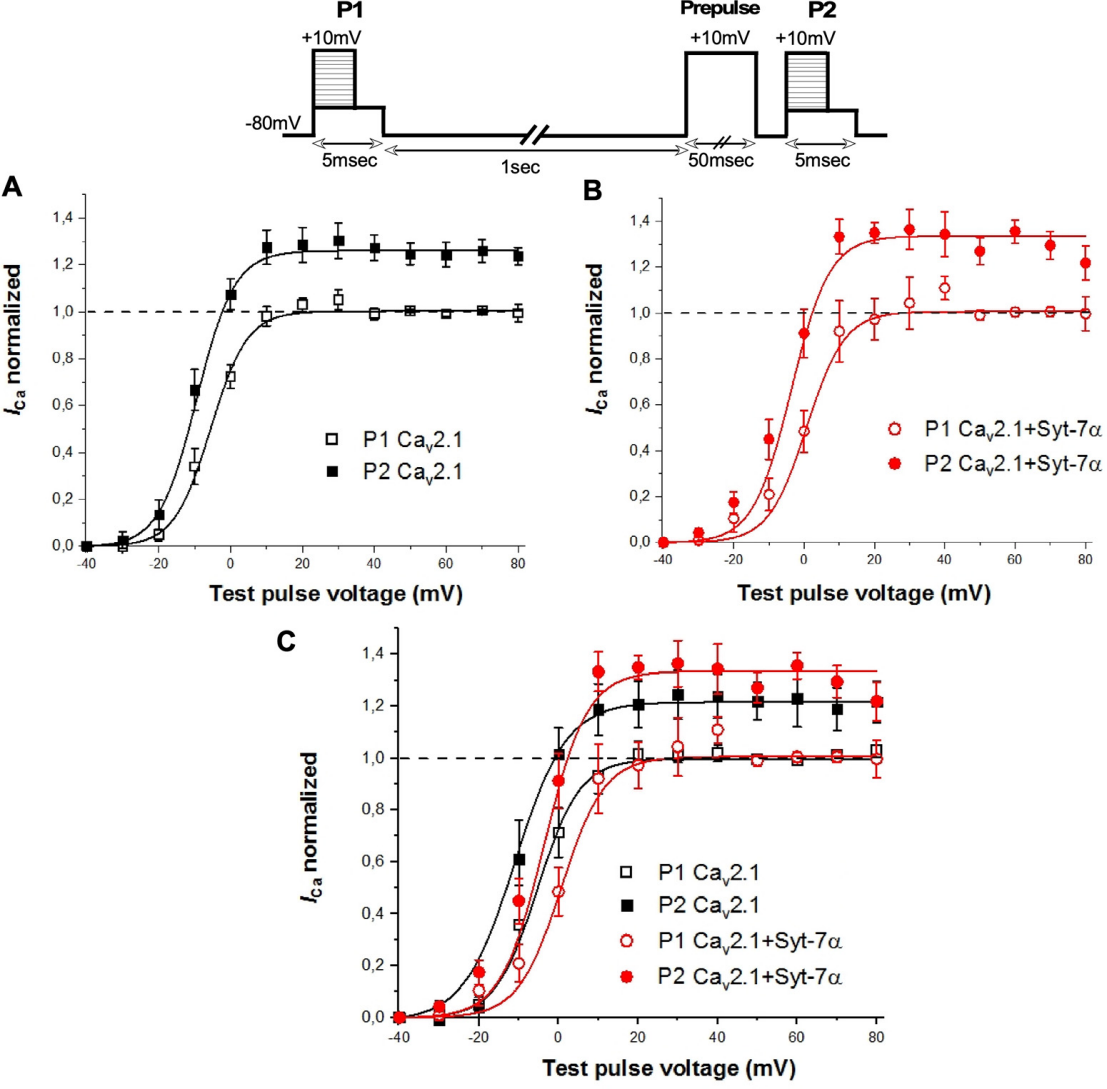
Effect of Syt-7α prepulse facilitation of Ca_v_2.1 channel at physiological levels. Inset, Voltage protocol. Currents recorded with 2 mm extracellular Ca^2+^ and 0.5 mm EGTA in the intracellular recording solution were elicited by test pulses to +10 mV before (P1) and 5 ms after (P2) 10-mV conditioning prepulses of the indicated durations. ***A*-*C***, Effect of Syt-7α on facilitation as a function of prepulse voltage. Facilitation was obtained by normalizing the peak current from P2 to that from P1. Single-exponential fits of the data are shown. Data are represented as mean ± SEM.

We also expressed Ca_v_2.1 channels without or with Syt-7 and measured the voltage dependence of activation of the resulting Ca^2+^ currents (Fig. 6). In this paired-pulse protocol described in Figure 6, inset, voltages were varied from −40 to +80 mV in both pulses P1 and P2 following a constant prepulse voltage of −80 to +10 mV before P2. Previous studies (Lee et al., 1999, 2000) showed that this protocol induced facilitation of Ca_v_2.1 channels. As shown in Figure 6*A–C*, Syt-7α significantly increased *I*_Ca_ across the positive voltage range and increased maximum facilitation at potentials of +40 mV and higher in the presence of 10 mm Ca^2+^. Syt-7α induced a significant ∼5- to 15-mV positive shift in the voltage dependence of Ca_v_2.1 activation, as observed by comparing the half-activation voltage (V_50_) at P1 (V_50_ = 14.28 ± 3.28, *n* = 7, *p* = 0.002) versus control cells (V_50_ = 3.96 ± 1.72, *n* = 19). During pulse P2, Syt-7α induced a significant ∼3.6- to 11-mV positive shift in the voltage dependence of Ca_v_2.1 activation (V_50_ = 7.96 ± 2.3, *n* = 7, *p* = 0.02) versus control cells (V_50_ = 0.7 ± 1.4, *n* = 19; Fig. 6*D*). This Syt-7α effect was also observed at physiological Ca^2+^ levels, where both facilitation amplitude and the positive shift in voltage dependence of Ca_v_2.1 activation were evident (Fig. 7). Together, these results show that Syt-7α induces strong facilitation of Ca^2+^ currents at membrane potentials in the range of the peak of action potentials (∼0 to +40 mV).

### Differential modulation of Ca_v_2.1 by isoforms of Syt-7

**Figure 8.**
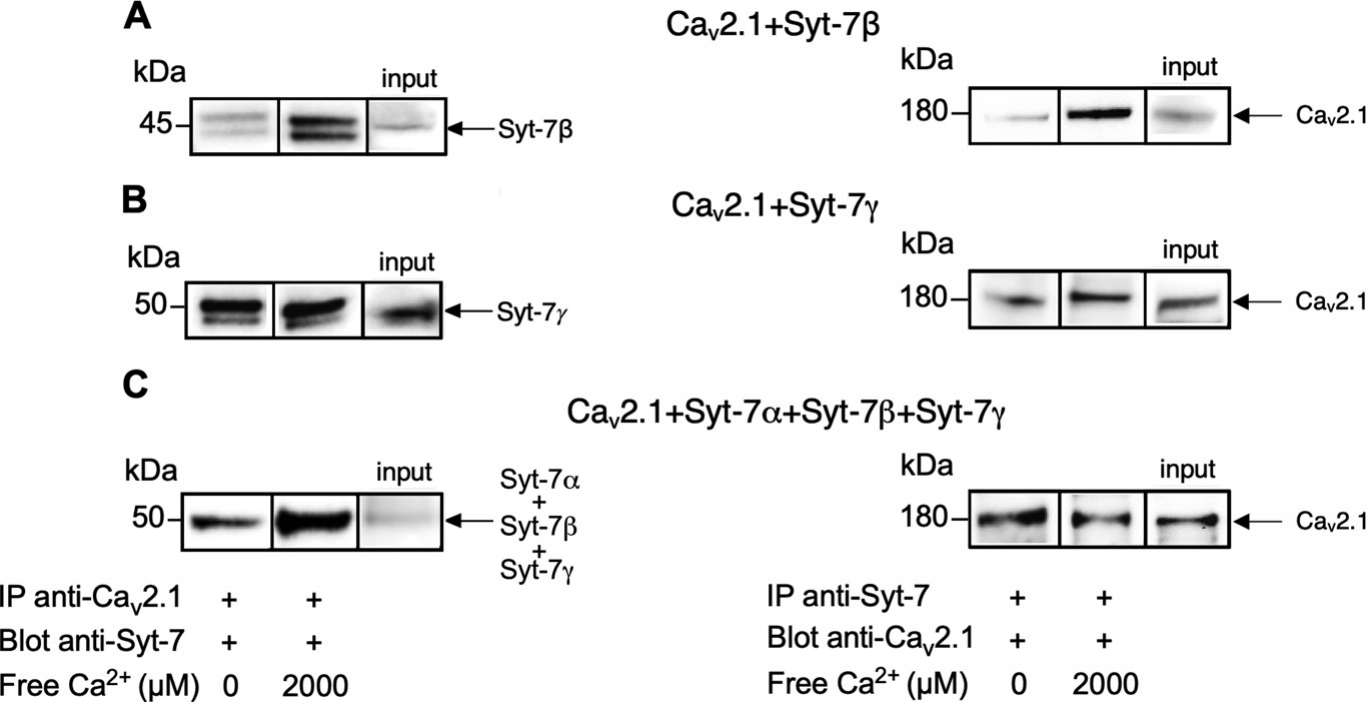
Syt-7 isoforms α, β, and γ co-immunoprecipitate with Ca_v_2.1 channels. ***A***, Left, In transfected tsA-201 cells, co-immunoprecipitation experiments show the binding of Syt-7β and α_1A_ subunit of Ca_v_2.1. Ca_v_2.1 channels were immunoprecipitated with an anti-Ca_v_2.1 antibody. The immunoprecipitates were resolved by SDS/PAGE and immunoblotted with antibody against Syt-7. Right, The reverse experiment where Syt-7β were immunoprecipitated with an anti-Syt antibody and blotted with anti-Ca_v_2.1 antibody. ***B***, Left, In tsA-201 cells co-transfected with the α_1A_ subunit of Ca_v_2.1 channels together with Syt-7γ, Ca_v_2.1 channels were immunoprecipitated with an anti-Ca_v_2.1 antibody and blotted with anti-Syt antibody. Right, Reverse experiment where Syt-7γ were immunoprecipitated with an anti-Syt antibody and blotted with anti-Ca_v_2.1 antibody. ***C***, Left, In tsA-201 cells co-transfected with the α_1A_ subunit of Ca_v_2.1 channels along with Syt-7α, β, and γ, Ca_v_2.1 channels were immunoprecipitated with an anti-Ca_v_2.1 antibody and blotted with anti-Syt antibody. Right, Reverse experiment where the three isoforms of Syt-7 were immunoprecipitated with an anti-Syt antibody and blotted with anti-Ca_v_2.1 antibody. Because different Ca^2+^ concentrations were used in co-immunoprecipitation experiments, segments from different immunoblots were spliced together to show specific comparisons. Those protein bands are delineated for clarification. The immunoblots presented here are representative of at least three experiments for each co-immunoprecipitation or immunoblot. Whole-cell voltage clamp experiments show that Ca_v_2.1 expressed alone gives similar peak Ba^2+^ and Ca^2+^ currents as Ca_v_2.1 + Syt7-αβγ (Extended Data Fig. 8-1).

Among the three Ca_v_2 subfamily members, only the Ca_v_2.1 channel supports short-term synaptic facilitation (Inchauspe et al., 2004); however, Syt-7 isoforms may have subtype-specific modulatory effects on Ca_v_2.1. Three splice variants of Syt-7 exist in mouse and human: the major form Syt-7α and two minor forms, Syt-7β and Syt-7γ (Fukuda et al., 2002). Syt-7β and Syt-7γ contain additional 44 and 116 amino acids, respectively, in the connecting segment between their transmembrane domain and the cytoplasmic C2 Ca^2+^-binding domain (Fukuda et al., 2002). Both the β and γ isoforms of Syt-7 were bound to Ca_v_2.1 channels in extracts of transfected tsA-201 cells to a similar extent as Syt-7α, as indicated by co-immunoprecipitation with anti-Ca_v_2.1 antibodies and immunoblotting with isoform-specific anti-Syt-7 antibodies (Fig. 8*A–C*, left). In complementary experiments, Ca_v_2.1 was co-immunoprecipitated from transfected tsA-201 cells with antibodies against Syt-7β and Syt-7γ (Fig. 8*A–C*, right). The consistent results in these two complementary immunoprecipitation protocols indicate that these protein interactions are specifically detected independent of the antibodies used for immunoblotting. Co-expression of Syt-7α, Syt-7β, and Syt-7γ together with Ca_v_2.1 channels did not have significant effects on the peak amplitude of either Ba^2+^ or Ca^2+^ currents in comparison to expression of Syt-7α alone (Extended Data Fig. 8-1). Together, these experiments indicate that all three Syt-7 isoforms bind to Ca_v_2.1 channels in transfected cells without significantly altering their level of functional expression.

10.1523/ENEURO.0081-22.2022.f8-1 Extended Data Figure 8-1 Effects of Syt-7 isoforms on peak Ba2+ and Ca2+ currents. IV relationships using depolarizing steps from –80 to +60 mV for 1 s. A, Cav2.1 alone and Cav2.1 + Syt7-α using 10 mm Ba2+ as permeant ion. B, Cav2.1 alone and Cav2.1 + Syt7-αβγ using 10 mm Ba2+ as permeant ion. C, Cav2.1 alone and Cav2.1 + Syt7-α using 10 mm Ca2+ as permeant ion. D, Cav2.1 alone and Cav2.1 + Syt7-αβγ using 10 mm Ca2+ as permeant ion. Data are represented as mean ± SEM. Download Figure 8-1, TIF file.

**Figure 9.**
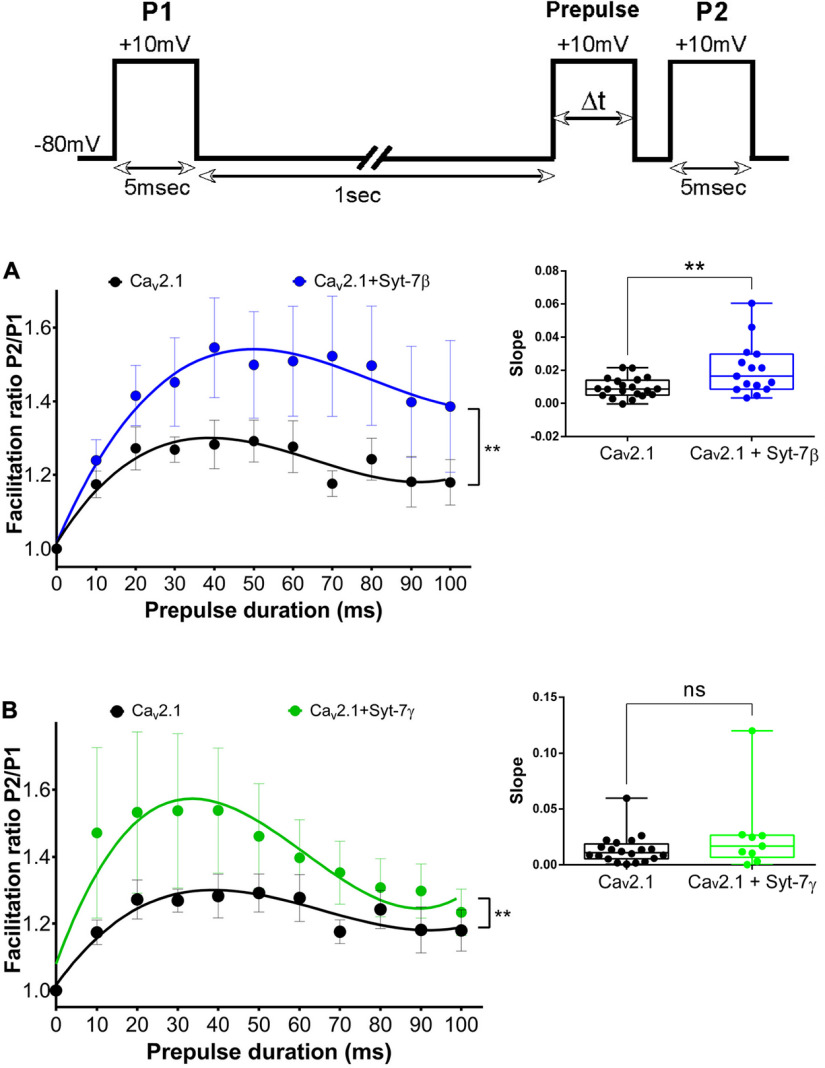
Syt-7β and Syt-7γ differentially modulate facilitation of Ca_v_2.1 channels. Inset, Pulse protocol. Currents recorded with 10 mm extracellular Ca^2+^ and 0.5 mm EGTA in the intracellular recording solution were elicited by test pulses to +10 mV before (P1) and 5 ms after (P2) 10-mV conditioning prepulses of the indicated durations. ***A***, Left, Syt-7β increases the facilitation ratio with increasing prepulse duration. Right, Syt-7β accelerates the onset of facilitation as a function of prepulse duration. ***B***, Left, Syt-7γ increases the facilitation ratio with increasing prepulse duration. Right, Syt-7γ does not accelerate the onset of facilitation as a function of prepulse duration. Facilitation was obtained by normalizing the peak current from P2 to that from P1. Single-exponential fits of the data are shown. Data are represented as mean ± SEM additional experiments with different pulse protocols provide additional information on the effects of Syt-7β and Syt-7γ on facilitation of Ca_v_2.1 channels (Extended Data Fig. 9-1).

We investigated the effects of co-expression with Syt-7β and Syt-7γ on facilitation of Ca_v_2.1 channels with 10 mm CaCl_2_ in the extracellular solution. Syt-7β increased the facilitation ratio of Ca_v_2.1 channels (*p* < 0.01; Fig. 9*A*) and accelerated the rate of facilitation, as demonstrated by the significant increase in the slope in cells co-transfected with Ca_v_2.1 plus Syt-7β (slope = 0.02 ± 0.004 ms^−1^; *n* = 15, *p* = 0.006) compared with control cells (slope = 0.009 ± 0.001 ms^−1^; *n* = 20; Fig. 9*A*, inset). However, these effects were substantially smaller than with co-expression of Syt-7α (Fig. 2). Co-expression of Syt-7γ also increased the peak level of facilitation (*p* < 0.01) to a lesser degree than Syt-7α (Fig. 9*B*), and it showed only a trend toward a significant increase in facilitation rate (slope = 0.03 ± 0.01 ms^−1^; *n* = 9, *p* = 0.16, ns) compared with control cells (slope = 0.01 ± 0.003 ms^−1^; *n* = 20; Fig. 9*B*, inset). To determine whether Syt-7β and Syt-7γ can compete effectively with Syt-7α, we co-expressed all three Syt-7 isoforms and measured Ca^2+^-dependent facilitation (Extended Data Fig. 9-1). We found that co-expression Syt-7β and Syt-7γ together with Syt-7α reduced the strong increase in the rate and extent of facilitation observed with Syt-7α alone (Extended Data Fig. 9-1, red). These results are consistent with the conclusion that Syt-7β and Syt-7γ effectively compete for occupancy of the synprint site and alter modulation of Ca_v_2.1 by Syt-7α. Evidently, replacement of Syt-7α with either Syt-7β or Syt-7γ at the synprint site would reduce facilitation of Ca_v_2.1 channels.

10.1523/ENEURO.0081-22.2022.f9-1 Extended Data Figure 9-1 Effects of Syt-7 isoforms on facilitation of Cav2.1 channels. Inset, Pulse protocol. Currents recorded with 10 mm extracellular Ca2+ and 0.5 mm EGTA in the intracellular recording solution were elicited by test pulses to +10 mV before (P1) and 5 ms after (P2) 10-mV preconditioning prepulses of the indicated durations. Main panel, Effect of Syt-7 isoforms on facilitation as a function of prepulse duration. Facilitation was obtained by normalizing the peak current from P2 to that from P1. Single-exponential fits of the data are shown. Data are represented as mean ± SEM. Download Figure 9-1, TIF file.

**Figure 10.**
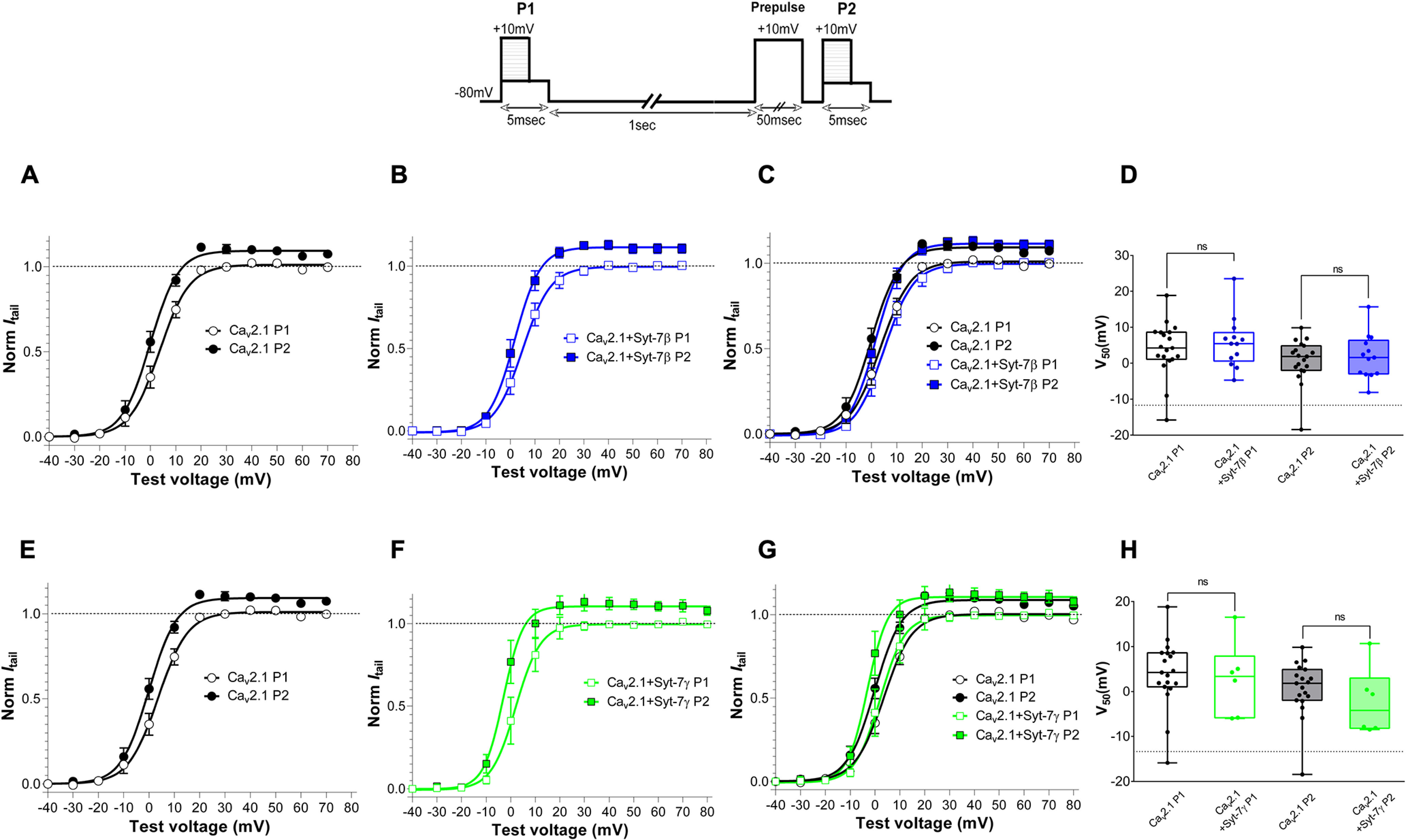
Syt-7β and Syt-7γ differentially modulate prepulse facilitation of Ca_v_2.1 channels. Inset, Pulse protocol. Currents recorded with 10 mm extracellular Ca^2+^ and 0.5 mm EGTA in the intracellular recording solution were elicited by test pulses to +10 mV before (P1) and 5 ms after (P2) 10-mV conditioning prepulses of the indicated durations. Facilitation was obtained by normalizing the peak current from P2 to that from P1. Single-exponential fits of the data are shown. ***A***, Ca_v_2.1 alone. ***B***, Ca_v_2.1 with Syt-7γ. ***C***, Overlay of panels ***A***, ***B***. ***D***, V_50_ values for results in panel ***C***. ***E***, Ca_v_2.1 alone. ***F***, Ca_v_2.1 with Syt-7γ. ***G***, overlay of panels ***D***, ***E***. ***H***, V_50_ values from panel ***G***. Data are represented as mean ± SEM additional experiments with different pulse protocols provide additional information on the effects of Syt-7β and Syt-7γ on the voltage dependence of facilitation of Ca_v_2.1 channels (Extended Data Fig. 10-1).

In experiments testing the effect of a depolarizing prepulse on the voltage dependence of activation, neither Syt-7β (Fig. 10*A–C*) nor Syt-7γ (Fig. 10*E–G*) increased the maximum prepulse facilitation of Ca_v_2.1 at positive membrane potentials in contrast to Syt-7α. Similarly, neither Syt-7β (Fig. 10*A–D*) nor Syt-7γ (Fig. 10*E–H*) caused a significant shift in the voltage dependence of activation of Ca_v_2.1 channels, unlike Syt-7α (Fig. 10*C*,*D*). Co-expressing the three Syt-7 isoforms together caused a negative shift in the voltage dependence of activation following a depolarizing prepulse, in contrast to the positive shift in the voltage dependence of activation following a prepulse caused by co-expression of Syt-7α alone (Extended Data Fig. 10-1, red). All of these voltage-dependent activation curves are monophasic (Figs. 7, 10; Extended Data Fig. 10-1), consistent with stoichiometric binding of each Syt-7 isoform to Ca_v_2.1 resulting in complete shifts of the activation curves. Together, these results suggest a dominant effect of Syt-7β and Syt-7γ on the voltage dependence of activation in paired-pulse protocols in the presence of all three Syt-7 isoforms.

10.1523/ENEURO.0081-22.2022.f10-1 Extended Data Figure 10-1 Effects of Syt-7 isoforms on the voltage-dependent activation of Cav2.1 channels. Inset, Pulse protocol. Currents recorded with 10 mm extracellular Ca2+ and 0.5 mm EGTA in the intracellular recording solution were elicited by test pulses to +10 mV before (P1) and 5 ms after (P2) 10-mV conditioning prepulses of the indicated durations. Main panel, Facilitation was calculated by normalizing the peak current from P2 to that from P1 for the expressed constructs indicated. Single-exponential fits of the data are shown. Data are represented as mean ± SEM. Note that the activation curve for Cav2.1 + Syt-7α alone (gray, dotted curve) is positively shifted with respect to Cav2.1 alone (black), whereas Cav2.1 + Syt-7αβγ (red) is negatively shifted with respect to Cav2.1 + Syt-7α (gray, dotted curve). Download Figure 10-1, TIF file.

**Figure 11.**
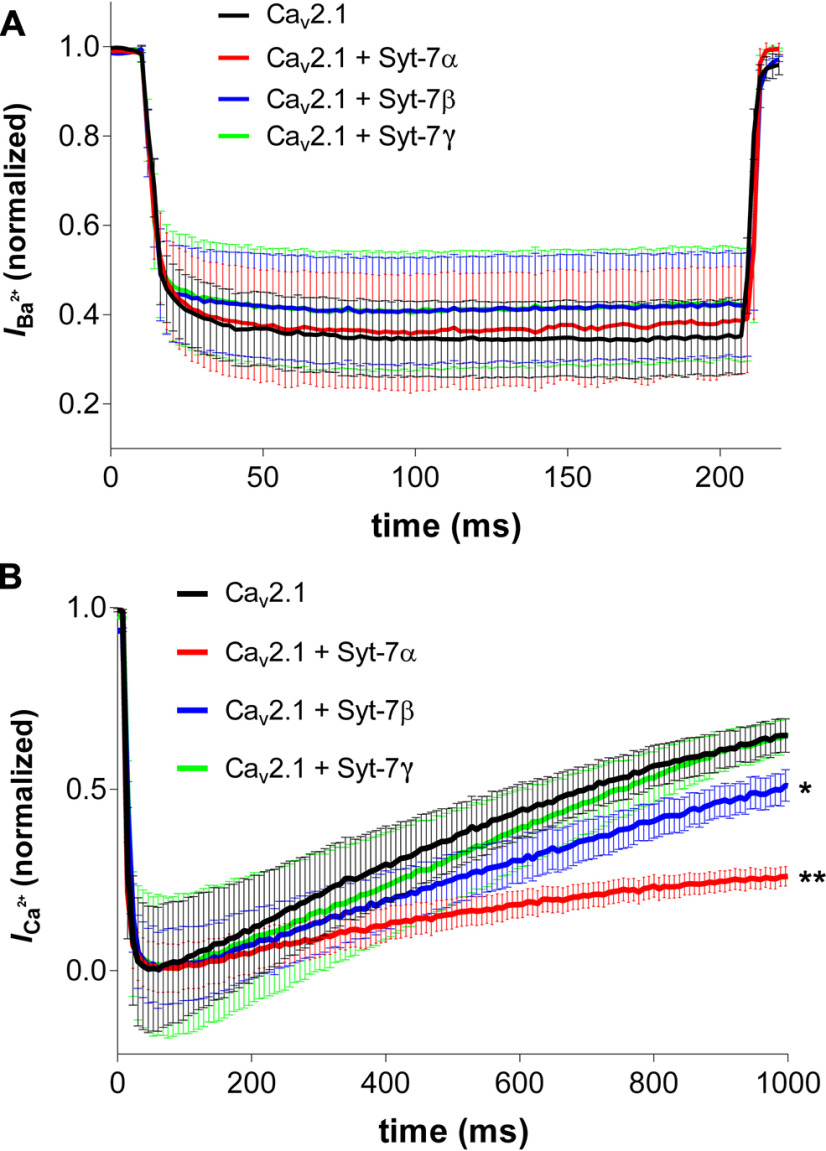
Syt-7 isoforms differentially modulate Ca^2+^-dependent inactivation of Ca_v_2.1 channels. Ca_v_2.1 currents were elicited by depolarizing from a holding potential of −80 mV to a test potential of +10 mV. ***A***, Time courses (200 ms) of I_Ba_ with 10 mm Ba^2+^ as a permeant cation. ***B***, Time courses (1000 ms) of I_Ca_ with 10 mm Ca^2+^ as permeant ion. Syt-7α and Syt-7β significantly slowed inactivation of the Ca_v_2.1 channel in the presence of 10 mm Ca^2+^, whereas Syt-7γ had no effect. Data are represented as mean ± SEM.

In addition to their differential effects on Ca^2+^-dependent facilitation, co-expression of Syt-7β or Syt-7γ also had different effects on Ca^2+^-dependent inactivation of Ca_v_2.1 channels compared with Syt-7α (Fig. 11). In the presence of 10 mm Ba^2+^ as the permeant extracellular cation, Ca_v_2.1 channels activated rapidly and did not inactivate significantly in 200-ms depolarizing pulses when co-expressed with any of the Syt-7 isoforms (Fig. 11*A*). In contrast, in the presence of 10 mm Ca^2+^ as permeant ion, Ca_v_2.1 channels inactivated with a time constant of ∼600 ms through their Ca^2+^/CaM-dependent inactivation mechanism (Fig. 11*B*, black). Strikingly, co-expression of Syt-7α substantially slowed Ca^2+^-dependent inactivation (Fig. 11*B*, red), whereas co-expression of Syt-7β had a smaller effect (Fig. 11*B*, blue) and co-expression of Syt-7γ had no effect on Ca^2+^-dependent inactivation (Fig. 11*B*, green). These results indicate that replacement of Syt-7α with either Syt-7β or Syt-7γ at the synprint site would decrease Ca^2+^ entry in single depolarizations by preventing the inhibition of Ca^2+^-dependent inactivation of Ca_v_2.1 channels induced by Syt-7α (Fig. 11), and at the same time would reduce prolonged Ca^2+^ entry by decreasing the enhanced facilitation of Ca_v_2.1 channels caused by Syt-7α during repetitive depolarizations (Fig. 9). This parallel modulation of Ca^2+^ entry by single depolarizations plus trains of depolarizations would have a potent impact on synaptic transmission. Altogether, these results indicate that the two minor Syt-7 isoforms bind to Ca_v_2.1 channels in cellular context without altering functional expression of Ca_v_2.1. However, co-expression of Syt-7β and Syt-7γ can partially reverse the functional effects of Syt-7α on facilitation (Extended Data Fig. 9-1), the voltage dependence of activation following a depolarizing prepulse (Extended Data Fig. 10-1), and the rate of Ca^2+^-dependent inactivation (Fig. 11). The differential actions of the three isoforms of Syt-7 provide a rich panoply of modulatory effects on Ca_v_2.1 channel activation, facilitation, and inactivation that would have a strong influence on synaptic transmission.

## Discussion

### Syt-7 modulates Ca_v_2.1 channels through binding to the synprint site

In presynaptic nerve terminals, Ca_v_2.1 and Ca_v_2.2 channels associate with SNARE proteins and a large number of other presynaptic proteins (Khanna et al., 2007; Müller et al., 2010; Nanou and Catterall, 2018). SNARE proteins interact with the synprint site located in the intracellular loop between domains II and III (Sheng et al., 1994, 1996; Rettig et al., 1996), which is thought to play an important role in the incorporation of Ca_v_2.1 channels into the synaptic vesicle fusion machinery and regulation of their function (Mochida et al., 2003b; Szabo et al., 2006). Ca^2+^ influx through Ca_v_2.1 channels is a crucial step in triggering Ca^2+^-dependent exocytosis of neurotransmitter vesicles (Olivera et al., 1994; Dunlap et al., 1995). Previous studies showed that the fast Ca^2+^ sensor Syt-1 binds to the synprint site of both Ca_v_2.1 and Ca_v_2.2 channels (Sakurai et al., 1996; Sheng et al., 1996; Charvin et al., 1997; Kim and Catterall, 1997). These protein interactions are likely to modulate the rapid, synchronous component of neurotransmitter release mediated by Syt-1 (Bacaj et al., 2013).

In contrast to these extensive studies of SNARE proteins and the fast Ca^2+^ sensor Syt-1, the slow, high-affinity Ca^2+^ sensor Syt-7 is unique in mediating synaptic facilitation (Jackman et al., 2016; Nanou et al., 2016a; Turecek and Regehr, 2018) and asynchronous release (Bacaj et al., 2013; Turecek and Regehr, 2018), but its interactions with Ca_v_2.1 channels had not previously been investigated. Our results presented here show that Syt-7 binds to the synprint site of Ca_v_2.1 channels *in vivo* in mouse brain membranes, *in vitro* in transfected cells, and in solution in protein-interaction experiments. Unexpectedly, in contrast to Syt-1, our results provide evidence that Syt-7 modulates Ca2^+^-dependent facilitation and inactivation (CDI) of Ca_v_2.1 channels, which are implicated in short-term forms of synaptic plasticity, including synaptic facilitation and the rapid phase of synaptic depression (Lee et al., 2000, 2002; Mochida et al., 2008; Nanou et al., 2016a, b, 2018). Direct interaction of Syt-7 and Ca_v_2.1 as shown here may contribute to short-term synaptic facilitation, in which both of these interacting protein partners are thought to play essential roles.

### Syt-7 isoforms differentially enhance facilitation of Ca_v_2.1 channels

In cells expressing Ca_v_2.1 channels, we consistently observed Ca^2+^-dependent facilitation of the Ca^2+^ current, as reported previously (Lee et al., 1999, 2000, 2003). In the presence of Syt-7α, both the rate and extent of facilitation of Ca_v_2.1 channels were increased, and the rate of decay of facilitation was also accelerated. These results suggest that expression of Syt-7α in presynaptic terminals *in vivo* would enhance Ca^2+^-dependent facilitation and sharpen the time-dependent peak of facilitation of Ca_v_2.1 channels. Syt-7β and Syt-7γ also bound to the synprint site. However, compared with Syt-7α, Syt-7β, and Syt-7γ had lesser effects on facilitation in response to voltage steps and did not shift the voltage dependence of prepulse facilitation. Differential expression of these Syt-7 isoforms could confer cell-specific regulation via interactions with Ca_v_2.1 channels and other regulatory targets.

### Syt-7 isoforms differentially modulate inactivation of Ca_v_2.1 channels

In our depolarizing step protocols, none of the Syt-7 isoforms had any significant effect on the peak amplitude of Ba^2+^ current. However, our data show that Syt-7α significantly increased Ca^2+^-dependent inactivation of the Ca^2+^ channel, which would oppose facilitation. Syt-7β and Syt-7γ had lesser effects. The combination of increased facilitation followed by increased inactivation induced by Syt-7α would have the overall effect of sharpening the peak of the presynaptic calcium current to allow effective facilitation of repetitive rounds of neurotransmitter release. Syt-7β and Syt-7γ would bind to the synprint site of Ca_v_2.1 channels but induce lesser functional effects.

### Comparison with regulation by CaM-like calcium sensor proteins

Our work characterizes an unexpected form of regulation of P/Q-type current conducted by Ca_v_2.1 channels by the high affinity Ca^2+^ sensor Syt-7 through a direct interaction with the synprint site. Interaction of Ca_v_2.1 with Syt-7 may enhance facilitation of presynaptic Ca^2+^ current and thereby play a role in triggering activation of the Ca^2+^-dependent exocytosis machinery, including the SNARE proteins. These effects would be dependent on the isoform of Syt-7 that is expressed in different cells and synapses. In previous experiments, CaM has been shown to regulate Ca_v_2.1 channel activity, inducing increased facilitation and increased Ca^2+^-dependent inactivation, dependent on the local Ca^2+^ concentration (Lee et al., 1999, 2000; DeMaria et al., 2001). Our results further show that Ca^2+^-dependent inactivation of Ca_v_2.1 channels is modulated by Syt-7 in an isoform-dependent manner. In presynaptic nerve terminals, these changes in both the Ca^2+^ entry in response to single action potentials plus trains of action potentials would substantially alter the encoding properties of synaptic transmission.

Other neuronal Ca^2+^ sensor proteins related to CaM are expressed in the central nervous system, including Ca^2+^ binding protein-1 (CaBP-1), visinin-like protein-2 (VILIP-2), and neuronal Ca^2+^ sensor-1 (NCS-1). These Ca^2+^ sensor proteins displace CaM from the C-terminal domain of Ca_v_2.1 and modify short-term synaptic facilitation and rapid synaptic depression (Nanou and Catterall, 2018). It will be interesting to further investigate how these two distinct regulatory mechanisms mediated by Syt-7 and Ca^2+^ sensor proteins converge on the Ca_v_2.1 channel on the millisecond time frame of short-term synaptic plasticity.

In conclusion, our work characterizes a novel form of regulation of P/Q-type Ca_v_2.1 channels by the high affinity Ca^2+^ sensor Syt-7 through direct interaction with the synprint site. Ca_v_2.1/Syt-7 interaction potentiates facilitation of Ca^2+^ current and may play a role in triggering Ca^2+^-dependent exocytosis along with other SNARE proteins. Syt-7 also modulates Ca^2+^/CaM-dependent inactivation. Understanding the mechanism by which Syt-7 isoforms enhance facilitation and modulate inactivation of Ca_v_2.1 channels in presynaptic terminals is a first step toward deciphering the complete picture of the role played by Syt-7 in the brain.

## Synthesis

Reviewing Editor: Kevin Wickman, University of Minnesota Decisions are customarily a result of the Reviewing Editor and the peer reviewers coming together and discussing their recommendations until a consensus is reached. When revisions are invited, a fact-based synthesis statement explaining their decision and outlining what is needed to prepare a revision will be listed below. The following reviewer(s) agreed to reveal their identity: NONE.

The authors have responded to most of the concerns raised during the initial round of review, and the revised manuscript includes helpful added details regarding experimental procedures (e.g., reagent IDs) as well as interesting co-expression experiments. There remains concern, however, regarding the immunoblotting data presented in Figures 1 and 8. Thin segments from blots were spliced together in many cases, and there are different degrees of background, saturation, and pixelation across the segments that are pasted together without any delineation. The sharp distinctions in appearance across segments suggest that they came from different blots or, alternatively, they came from same blot but with segment-specific digital processing. This approach is neither acknowledged nor justified in the manuscript or rebuttal, and it is expressly forbidden in some journals. In this case, it complicates comparison of bands and band intensities across experimental conditions. The experiments should be re-run in a manner that allows all samples to be run on the same blot and in adjacent lanes, to avoid any need for cutting/pasting blot segments together. At a minimum, the authors need to clearly delineate individual blot segments and provide reassurance that no segment-specific digital processing occurred. Related to all this, there is currently no mention in the manuscript that the immunoblotting data presented in these figures is representative of multiple attempts at the same experiment. Given the significance of these data to the manuscript conclusions, it is important to confirm that the outcomes were reproducible across multiple trials.
