# Supplementary figures and images for: Synaptotagmin-7 Enhances Facilitation of Cav2.1 Calcium Channels
Source: eNeuro. 2022 May 12;9(3):ENEURO.0081-22.2022. doi: 10.1523/ENEURO.0081-22.2022 (PMC9113918; doi:10.1523/ENEURO.0081-22.2022)

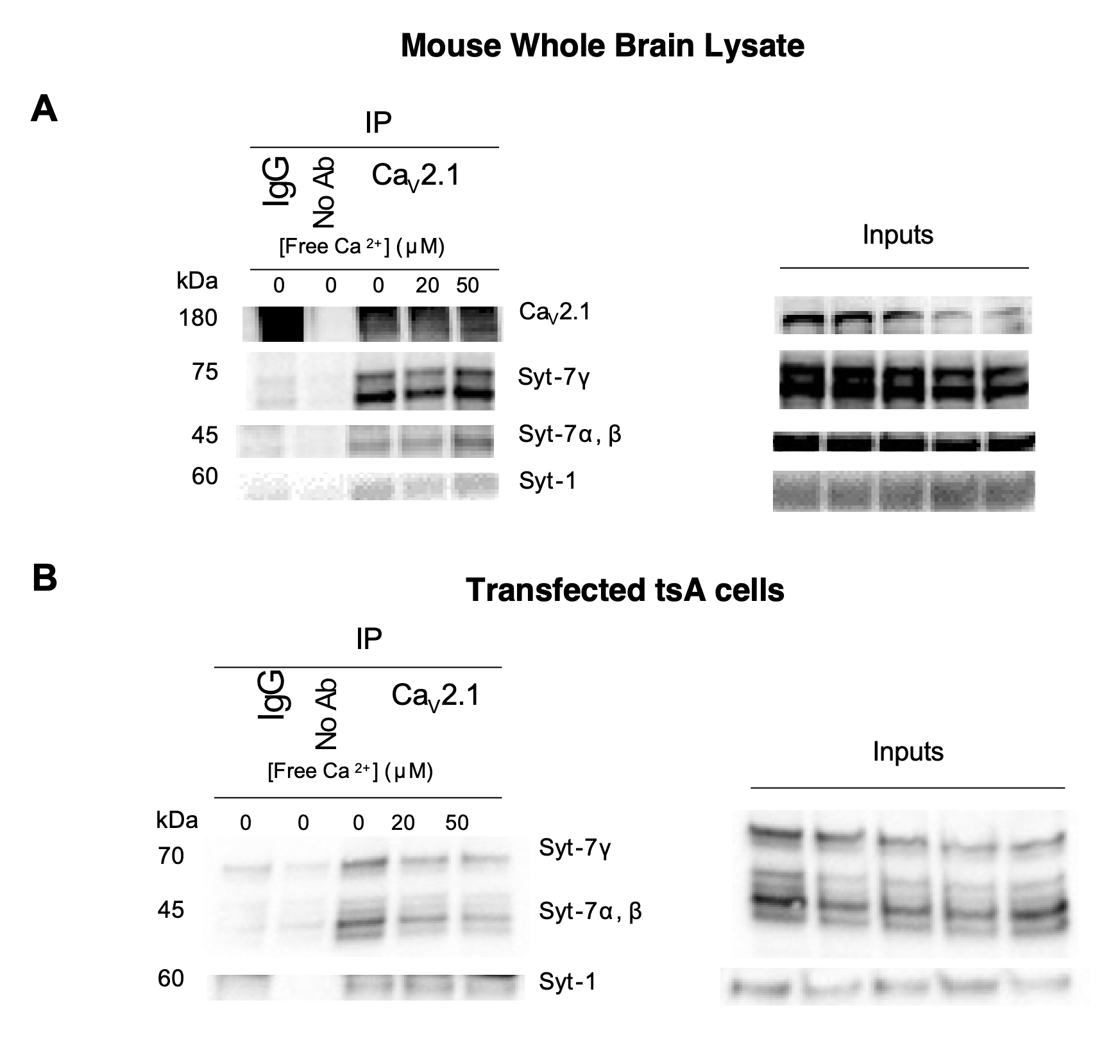

Supplement: Extended Data Figure 1-1 — Co-immunoprecipitation of Syt-7 and Syt-1 with Cav2.1 channels. A, In mouse brain lysate, Syt-7 proteins co-immunoprecipitated with Cav2.1 channels in three different buffered concentrations of free Ca2+. B, Co-immunoprecipitation of Syt-7 and Syt-1 with Cav2.1 from co-transfected tsA-201 cells. Syt-7 is pulled down by Cav2.1-specific antibodies in Cav2.1 and Syt-7 co-transfected tsA-201 cell lysates. Download Figure 1-1, TIF file. [file enu-eN-NWR-0081-22-s02.tif]

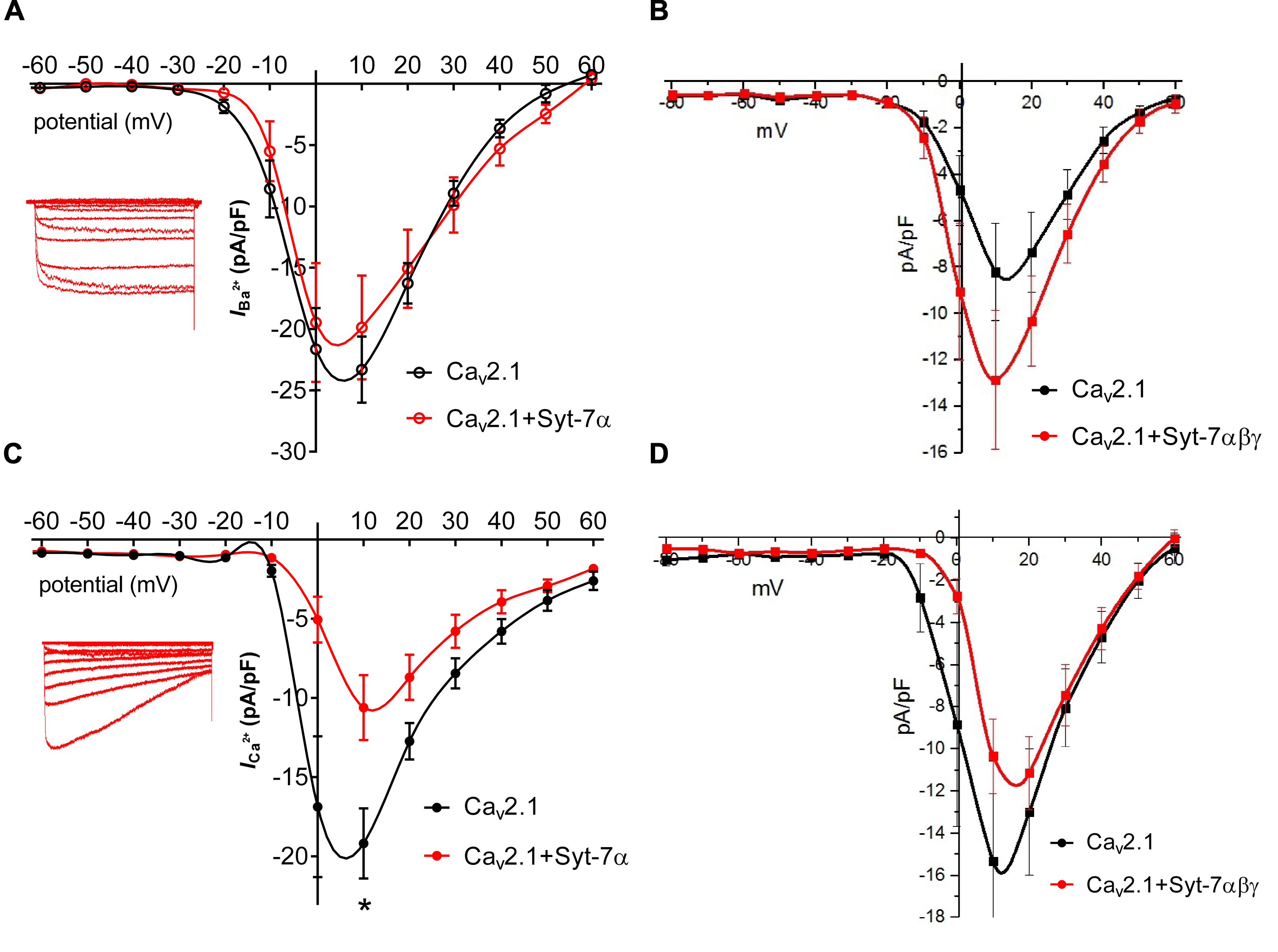

Supplement: Extended Data Figure 8-1 — Effects of Syt-7 isoforms on peak Ba2+ and Ca2+ currents. IV relationships using depolarizing steps from –80 to +60 mV for 1 s. A, Cav2.1 alone and Cav2.1 + Syt7-α using 10 mm Ba2+ as permeant ion. B, Cav2.1 alone and Cav2.1 + Syt7-αβγ using 10 mm Ba2+ as permeant ion. C, Cav2.1 alone and Cav2.1 + Syt7-α using 10 mm Ca2+ as permeant ion. D, Cav2.1 alone and Cav2.1 + Syt7-αβγ using 10 mm Ca2+ as permeant ion. Data are represented as mean ± SEM. Download Figure 8-1, TIF file. [file enu-eN-NWR-0081-22-s03.tif]

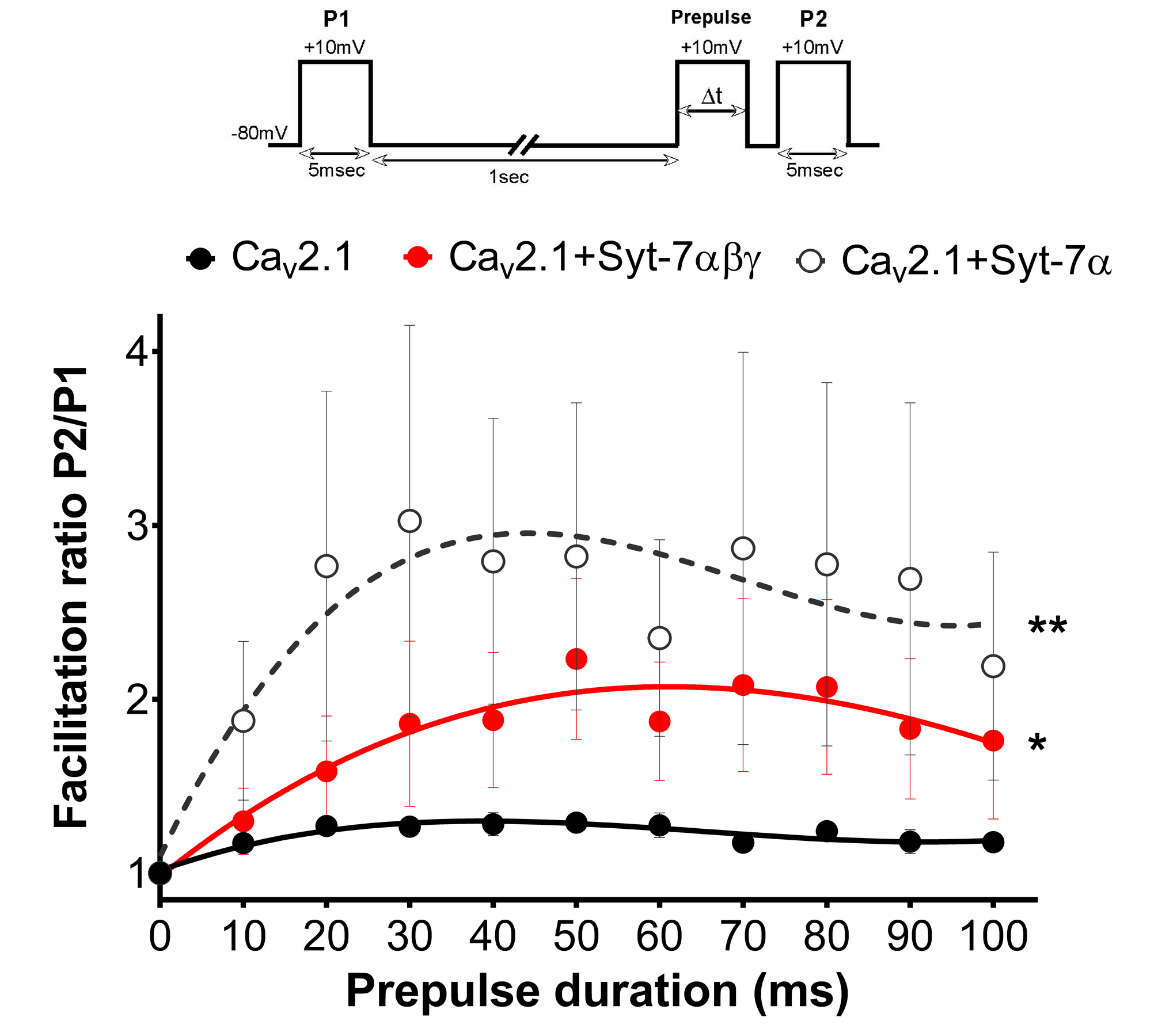

Supplement: Extended Data Figure 9-1 — Effects of Syt-7 isoforms on facilitation of Cav2.1 channels. Inset, Pulse protocol. Currents recorded with 10 mm extracellular Ca2+ and 0.5 mm EGTA in the intracellular recording solution were elicited by test pulses to +10 mV before (P1) and 5 ms after (P2) 10-mV preconditioning prepulses of the indicated durations. Main panel, Effect of Syt-7 isoforms on facilitation as a function of prepulse duration. Facilitation was obtained by normalizing the peak current from P2 to that from P1. Single-exponential fits of the data are shown. Data are represented as mean ± SEM. Download Figure 9-1, TIF file. [file enu-eN-NWR-0081-22-s04.tif]

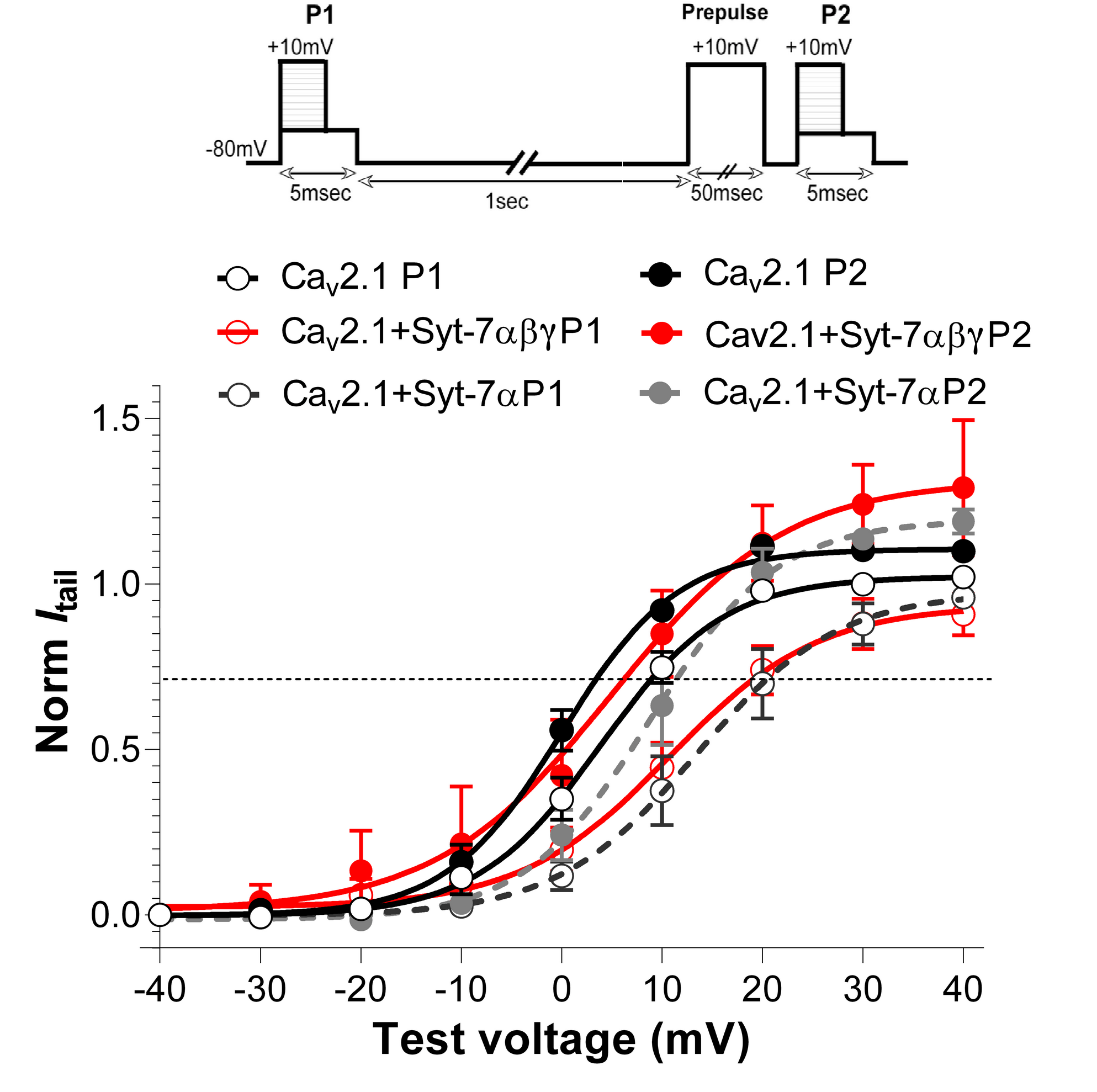

Supplement: Extended Data Figure 10-1 — Effects of Syt-7 isoforms on the voltage-dependent activation of Cav2.1 channels. Inset, Pulse protocol. Currents recorded with 10 mm extracellular Ca2+ and 0.5 mm EGTA in the intracellular recording solution were elicited by test pulses to +10 mV before (P1) and 5 ms after (P2) 10-mV conditioning prepulses of the indicated durations. Main panel, Facilitation was calculated by normalizing the peak current from P2 to that from P1 for the expressed constructs indicated. Single-exponential fits of the data are shown. Data are represented as mean ± SEM. Note that the activation curve for Cav2.1 + Syt-7α alone (gray, dotted curve) is positively shifted with respect to Cav2.1 alone (black), whereas Cav2.1 + Syt-7αβγ (red) is negatively shifted with respect to Cav2.1 + Syt-7α (gray, dotted curve). Download Figure 10-1, TIF file. [file enu-eN-NWR-0081-22-s05.tif]
